# A Voltammetric Perspective of Multi-Electron and Proton Transfer in Protein Redox Chemistry: Insights From Computational Analysis of *Escherichia coli* HypD Fourier Transformed Alternating Current Voltammetry

**DOI:** 10.3389/fchem.2021.672831

**Published:** 2021-06-14

**Authors:** Alister R. Dale-Evans, Martin J. Robinson, Henry O. Lloyd-Laney, David J. Gavaghan, Alan M. Bond, Alison Parkin

**Affiliations:** ^1^ Department of Computer Science, University of Oxford, Oxford, United Kingdom; ^2^ School of Chemistry, Monash University, Clayton, VIC, Australia; ^3^ Department of Chemistry, University of York, Heslington, United Kingdom

**Keywords:** protein electrochemistry, ac voltammetry, surface confined voltammetry, proton-coupled electron-transfer, *Escherichia coli* HypD, disulfide redox

## Abstract

This paper explores the impact of pH on the mechanism of reversible disulfide bond (CysS-SCys) reductive breaking and oxidative formation in *Escherichia coli* hydrogenase maturation factor HypD, a protein which forms a highly stable adsorbed film on a graphite electrode. To achieve this, low frequency (8.96 Hz) Fourier transformed alternating current voltammetric (FTACV) experimental data was used in combination with modelling approaches based on Butler-Volmer theory with a dual polynomial capacitance model, utilizing an automated two-step fitting process conducted within a Bayesian framework. We previously showed that at pH 6.0 the protein data is best modelled by a redox reaction of two separate, stepwise one-electron, one-proton transfers with slightly “crossed” apparent reduction potentials that incorporate electron and proton transfer terms ($E_{\text{app}2}^{0}$ > $E_{\text{app}1}^{0}$). Remarkably, rather than collapsing to a concerted two-electron redox reaction at more extreme pH, the same two-stepwise one-electron transfer model with $E_{\text{app}2}^{0}$ > $E_{\text{app}1}^{0}$ continues to provide the best fit to FTACV data measured across a proton concentration range from pH 4.0 to pH 9.0. A similar, small level of crossover in reversible potentials is also displayed in overall two-electron transitions in other proteins and enzymes, and this provides access to a small but finite amount of the one electron reduced intermediate state.

## Introduction

**FIGURE 1:**
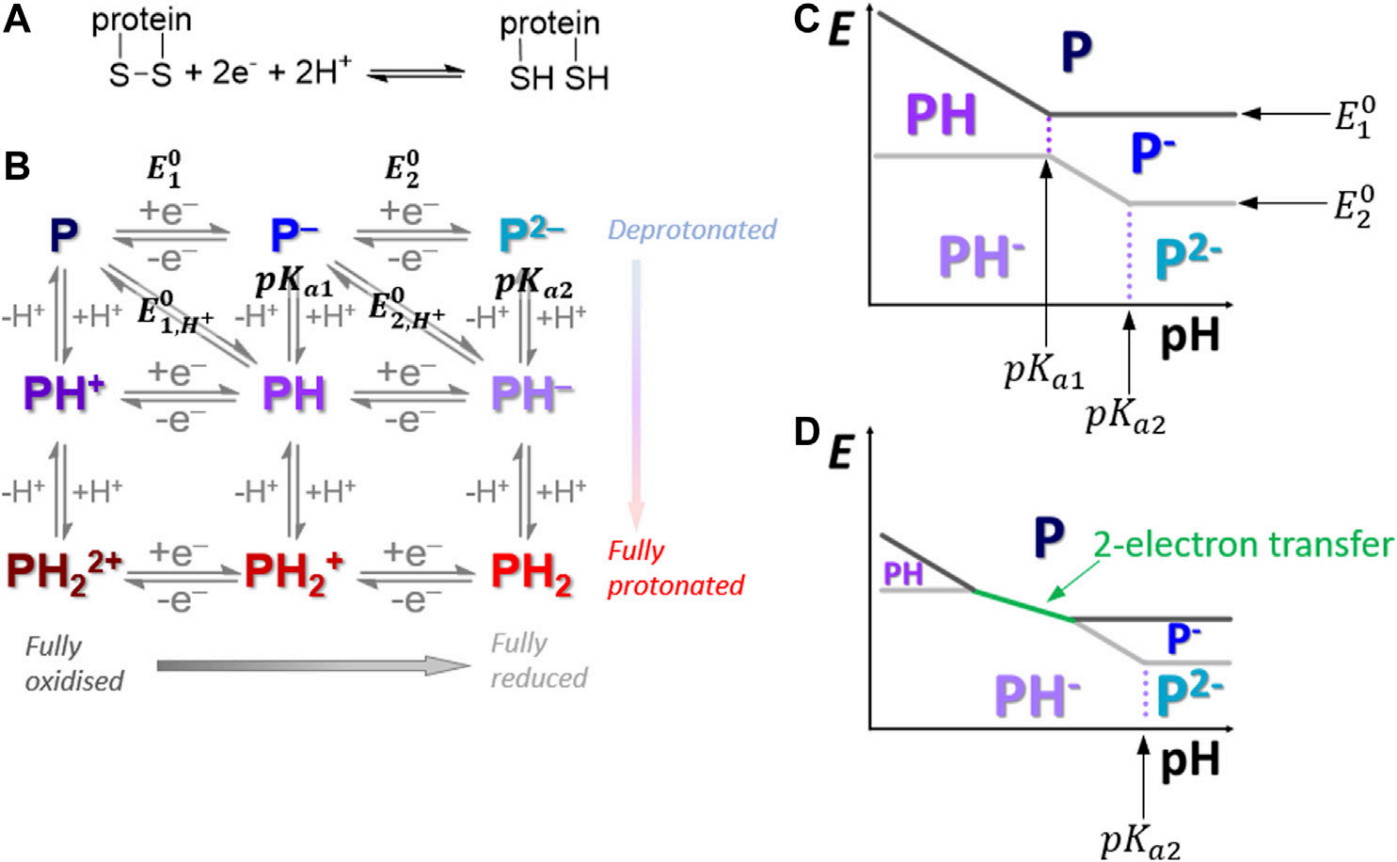
**(A)** Overall disulfide bond two-proton, two-electron oxidative-formation and reductive-cleavage reaction. **(B)** How the overall reaction separates into a square-scheme of vertical one-proton and horizontal one-electron transfers where “P” denotes the protein redox center. **(C,D)** Pourbaix diagrams depicting how pH can influence the reaction pathway across the top half of the Pourbaix diagram.

The redox chemistry of metalloenzymes and metalloproteins frequently occurs *via* multiple electron transfer events coupled to proton transfer (Hirst, 2006; Weinberg et al., 2012; Savéant and Costentin, 2019). For example, many of the enzymes which underpin photosynthesis and have application in artificial biological solar fuel production (i.e., Photosystem II, hydrogenases, and carbon monoxide dehydrogenases) rely on the efficient, concerted movement of protons and electrons to ensure product selectivity (Evans et al., 2019). Protein film electrochemistry (PFE) has been shown to be a powerful technique to probe the redox biochemistry of such proton-coupled electron transfer reactions (Armstrong et al., 1997; Hirst, 2006; Fourmond and Léger, 2017). As we have demonstrated previously (Adamson et al., 2017b), *Escherichia coli* hydrogenase maturation factor HypD (elsewhere “H2ase-MFHypD”), an enzyme which is important in the biosynthesis of hydrogenases (Nutschan et al., 2019), acts as a relatively simple example of such biological proton-coupled electron transfer redox chemistry. When H2ase-MFHypD was immobilized on a graphite electrode we could use both classical direct current cyclic voltammetry (DCV, involving a linear potential-time ramp) as well as large amplitude Fourier transform alternating current voltammetry (FTACV, utilizing a sine-wave plus linear-ramp voltage-time oscillation) to observe reversible disulfide bond reductive cleavage and oxidative formation (Supplementary Figure S1), a reaction that is considered to be a net two-proton, two-electron reaction at near-neutral pH (Figure 1A) (Adamson et al., 2017b).

An advantage of using FTACV over DCV in dynamic electrochemical measurements is that the higher order harmonic current-time responses (isolated by the sequence of Fourier transformation of the total current, band selection of current at a certain frequency, and inverse Fourier transformation) emphasize the response of fast electron transfer processes, i.e., current from rapid electron transfer processes can be separated from that arising from slower processes (Zhang and Bond, 2007; Adamson et al., 2017a). The isolation of fast electron transfer current from other contributions becomes particularly powerful when FTACV is applied to PFE (Adamson et al., 2017a; Zouraris et al., 2018). This is attributed to the fact that in many PFE studies when looking at the DC/fundamental harmonic current there is a low ratio of Faradaic signal from non-catalytic electron transfer processes relative to the capacitive background charging current. This arises because the large footprint of the protein molecule only allows a low surface coverage of redox active biomolecules to be present on the electrode surface (Hirst, 2006; Adamson et al., 2017a; Fourmond and Léger, 2017). As a result of these benefits, large amplitude FTACV is beginning to be adopted by the electrochemical community and is now being used in multiple laboratories (Zhang et al., 2018; Zouraris et al., 2018; Kumari and Adeloju, 2019; Zheng et al., 2019). In this study, as well as exploiting access to the much improved faradaic-to-charging current ratio, we specifically demonstrate the utility of the technique in distinguishing reaction mechanisms of the kind showcased initially in our previous work (Adamson et al., 2017b). Use of AC techniques to distinguish mechanisms that give rise to similar voltammetric data is an area of increasing research as shown by the recent use of square-wave voltammetry to distinguish between one and two-step reaction mechanisms (Gulaboski, 2019; Gulaboski and Mirceski, 2020; Guziejewski, 2020).

In this study, although H2ase-MFHypD serves as an extremely useful test system for developing our data analysis techniques for probing disulfide mechanisms, it is not possible to conclude if this redox-driven bond making/breaking is relevant to the physiological function of the enzyme (Adamson et al., 2017b; Nutschan et al., 2019). However, there are a multitude of proteins and enzymes where the disulfide chemistry is vitally important and *in vitro* electrochemical studies do provide a useful insight into the *in vivo* biological chemistry, as demonstrated by the comprehensive study by Bewley et al. (Bewley et al., 2015).

In our previous publication (Adamson et al., 2017b), PF-FTACV H2ase-MFHypD data collected at pH 6.0 was analyzed using automated solver methods to address the inverse problem, i.e., return a set of reaction model parameter values which generated a simulated dataset that provides a “best fit” to the experimental data, as quantified by an objective function that measured the least squares distance between the experimental and simulated data (Adamson et al., 2017b). Based on this analysis we concluded that at pH 6.0 solution conditions the overall H2ase-MFHypD redox reaction was most accurately modelled as two separate one-electron, one-proton steps with very similar associated reversible potentials (Adamson et al., 2017b). We therefore considered that at pH 6.0 the overall reaction proceeded from the top-left to the bottom-right of the square scheme shown in Figure 1B (Adamson et al., 2017b).

The best fit parameters for modelling H2ase-MFHypD FTACV at pH 6.0 and low frequency were consistent with the reaction proceeding under equilibrium conditions and the second electron transfer having an apparent reversible potential value (*E*_app_, a potential term relating to the equilibrium point of an electrochemical-chemical “EC” process comprising electron transfer and chemical steps) more positive than for the first process by about 10 mV (Laviron, 1980; Adamson et al., 2017b). This is broadly observed across many two-electron transfer biological processes, and generally the redox crossover between the separate potential values is sufficiently small to permit a finite potential window to exist in which the intermediate one-electron state can be detected, as predicted by the Nernst equation (Supplementary Figure S2) (Lopez-Tenes et al., 2014; Evans et al., 2019).

As in Figure 1B, in proton-coupled electron transfer reactions the formal potential associated with an overall proton-electron equilibrium reaction is often denoted a value of $E_{n,H^{+}}^{0}$ as it is derived from a combination of $E_{n}^{0}$, the potential associated with the *n*th electron-transfer, and $pK_{an}$, the equilibrium position of the associated protonation process (Laviron, 1980; Bond, 2002). As shown by Figures 1C,D, the origin of “crossed” one-proton, one-electron potential values, i.e., $E_{2,H^{+}}^{0}$, the equilibrium value for adding a second electron and a proton, being more positive than $E_{1,H^{+}}^{0}$, the equilibrium value for adding a first electron and a proton, can be rationalized by considering that although $E_{2}^{0}$ will be negative relative to $E_{1}^{0}$ (simplistically ascribed to the increased charge repulsion between the one-electron reduced state ${\text{P}}^{-}$ and the second electron relative to the oxidized “P” state and the first electron), if the species ${\text{P}}^{2-}$ is a stronger base than ${\text{P}}^{-}$ (i.e., $pK_{a2}$ > $pK_{a1}$, as is usually the case due to electrostatic attraction), then as solutions become more acidic the ordering of the potentials can change from $E_{2,H^{+}}^{0}$ more negative than $E_{1,H^{+}}^{0}$, to equal values and, ultimately, $E_{2,H^{+}}^{0}$ more positive than $E_{1,H^{+}}^{0}$, i.e., “crossed” potentials. At sufficiently high potential crossover, i.e., when $E_{2,H^{+}}^{0}$ is sufficiently more positive than $E_{1,H^{+}}^{0}$, the two-stepwise one-electron scenario is equivalent to the simultaneous two-electron transfer, as is shown by Figure 1D and the Nernst plots in Supplementary Figure S2. This is thoroughly described and derived in the seminal paper on a 9-member square scheme by Laviron (Laviron, 1983).

In our earlier work on H2ase-MFHypD (Adamson et al., 2017b), although we collected experimental data from pH 4.0 to pH 9.0 (Supplementary Figure S3), we did not explore the impact of proton availability on the conserved appropriateness of a stepwise two-sequential, one-electron transfer model, or on the tuning of the redox-potential crossover; this is the focus of the work presented here. We aim to establish if the same reaction model is appropriate under alkaline solution conditions (up to pH 9.0) and acidic solution conditions (down to pH 4.0) by comparing the accuracy with which a simulated dataset provides a “best fit” to the experimental data using either a two-stepwise one-electron transfer reaction model (*n*_*1*_ = *n*_2_ = 1) or a one-concerted two-electron transfer model (*n*_*3*_ = 2). We also explore if an equilibrium reaction model is appropriate across the entire pH range. Overall, we are therefore probing how proton availability in the bulk solution-phase impacts the mechanism of biological redox chemistry, as well as showcasing the power of FTACV in allowing differentiation between reaction models.

## Materials and Methods

### Electrochemistry Measurements

All data was collected in our previous study (Adamson et al., 2017b), all experiments were conducted at 25°C and all potentials are reported vs. the standard hydrogen electrode (SHE). An example of raw experimental data and verification of protein adherence can be seen in Supplementary Figure S11. In FTACV the input potential as a function of time, E(t), is formulated as Eq. 1.

$$E\left(t\right)=E_{\text{dc}}\left(t\right)+\text{Δ}E\text{sin}\left(\omega t+\eta \right).$$ (1)

The term $E_{\text{dc}}\left(t\right)$ describes dc contributions to the overall input potential. In all the experimental data shown the dc scan rate (*ν*) was 22.4 mV s^−1^. The second term, ΔEsin(ωt+η), describes the ac contribution to the FTACV experiment; ΔE is the amplitude of the sine wave and in all experimental data in the paper ΔE = 150 mV. The term ω denotes the angular frequency, where ω=2πf; f is the frequency of the sine wave and in all experimental data in the paper f = 8.96 Hz. Finally, the term η indicates the phase, the values of which are determined *via* fittings to the experimental data detailed below.

### Mathematical Model of the Faradaic Current

Modelling has been undertaken to calculate the Faradaic current output from the FTACV interrogation of a two-sequential one-electron transfer process described by Reaction 1 and Reaction 2 (referred to as the *n*_*1*_ = *n*_2_ = 1 process), and a concerted two-electron process, as given by Reaction 3 (elsewhere designated *n*_*3*_ = 2). Other researchers have published work on the experimental measurement and theoretical modelling of solution phase voltammetry of such electrochemical-electrochemical stepwise multiple electron transfer processes (Evans, 2008; Lopez-Tenes et al., 2014). We, and others, have published a number of analogous studies on surface confined species such as the H2ase-MFHypD system we describe here (Finklea, 2001; Lee et al., 2011; Stevenson et al., 2012; Robinson et al., 2018; Robinson et al., 2019).

$$X_{\left(surf\right)}+e^{-}\overset{k_{1}^{red}}{\underset{k_{1}^{ox}}{⇌}}Y_{\left(surf\right)}\;\left(E_{\mathrm{app}1}^{0},k_{\mathrm{app}1}^{0},\alpha_{\mathrm{app}1}^{0}\right)\text{Reaction 1}$$

$$Y_{\left(surf\right)}+e^{-}\overset{k_{2}^{red}}{\underset{k_{2}^{ox}}{⇌}}Z_{\left(surf\right)}\;\left(E_{\mathrm{app}2}^{0},k_{\mathrm{app}2}^{0},\alpha_{\mathrm{app}2}^{0}\right)\text{Reaction 2}$$

$$X_{\left(surf\right)}+2e^{-}\overset{k_{3}^{red}}{\underset{k_{3}^{ox}}{⇌}}Z_{\left(surf\right)}\;\left(E_{\mathrm{app}3}^{0},k_{\mathrm{app}3}^{0},\alpha_{\mathrm{app}3}^{0}\right)\text{Reaction 3}$$

It is assumed that protonation reactions accompanying the electron transfer are reversible (diffusion controlled) which allows modelling to be undertaken by combining the E^o^, K_a_ and pH terms of a reaction into an apparent $E_{app}^{0}$ value which is defined as the reversible potential. The reaction processes are treated as quasi-reversible with $k_{app}^{0}$, the electrode kinetics parameters describing the electron transfer rate constant at $E_{app}^{0}$, and the charge transfer coefficient $\alpha_{app}^{0}$ being added to the model *via* the Butler-Volmer relationship (Bard and Faulkner, 2001). The Faradaic current term is scaled by the total amount of H2ase-MFHypD on the electrode surface and therefore the parameter *Γ*, denoting the surface coverage per unit area of protein on the electrode, is also incorporated into the Faradaic current models with a value of 0.03 cm^2^ used to account for the geometric surface area of the electrode tip.

As detailed in the Results, an advantage of analyzing H2ase-MFHypD FTACV data compared to other proton coupled electron transfer protein systems is that we show that we are in a parameter regime where we do not have to incorporate kinetic dispersion into our simulation model; this avoids a substantial additional computational cost, as shown previously (Lloyd-Laney et al., 2021b). For simplicity, and as shown previously (Adamson et al., 2017b), we can also neglect a possible small contribution from thermodynamic dispersion (Léger et al., 2002; Morris et al., 2015); this is advantageous as it makes the data fitting computationally less demanding (and therefore faster), and it also decreases the number of models which must be compared (Lloyd-Laney et al., 2021b).

### Two-step Approach to Solving the Inverse Problem

To extract the reaction model parameter values which gave the “best fit” between the experimental electrochemical data and either a simulation of the two-sequential one-electron transfer model (*n*_*1*_ = *n*_2_ = 1) or a simulation of the consecutive two-electron transfer model (*n*_*3*_ = 2) a two-step process was used, based on that described in our previous work (Adamson et al., 2017b). This two-step process takes advantage of the fact that the total current recorded in an experiment is a sum of both the Faradaic current arising from the redox reaction under interrogation and non-Faradaic background capacitive charging current contributions.

First, using analysis in the time-domain, we determined the parameter values which gave the best fit between a simulated non-Faradaic capacitance current trace (see Supplementary Material for details of the third order polynomial “capacitance” model) and regions of the experimental current trace with little or no Faradaic contribution (Supplementary Figures S5, S6) by performing 10 optimizations per experimental dataset and recording the parameter value combination which gave the minimal sum of square difference between the simulated capacitance current and the experimental current. Amongst other parameters, this enabled the determination of “best fit” values for the uncompensated resistance, $R_{\text{u}}$. Secondly, using Fourier transformation to convert the total current trace into the frequency domain, full models were used to simulate both a non-Faradaic capacitance-current component using the pre-determined “capacitance” and $R_{\text{u}}$ parameter values, and find the “best fit” Faradaic reaction model parameters using either the model for two-stepwise one-electron transfers ($n_{1}=n_{2}=1$) or the model for one-concerted two-electron transfer ($n_{3}=2$). A phase parameter, η, and the protein density on the electrode surface, Γ, were also determined in this latter process. Again, 10 optimizations were performed for each experimental current trace and the “best fit” values were defined as those which gave a minimal value for an Euclidean distance objective function that measures the difference between simulated Fourier transformed current and Fourier transformed experimental current.

Full details of the mathematical model, as well as a more in-depth description of the optimization methods used to solve the inverse problem are provided in the Supplemantary Material (Gavaghan et al., 2018; Clerx et al., 2019; Harris et al., 2020; Gundry et al., 2021).

## Results

### Model Verification

The models and the inference methods used in this study were extensively verified based on synthetic data studies and the reproduction of results from the literature, as detailed in Verification of computational methods in the Supplementary Material.

### Two-Sequential One-Electron Transfer Reaction Model Vs. Concerted Two Electron Transfer Model

The two-step fitting process described in the Materials and Methods was used to extract “best fit” parameter values for the pH 4.0, 5.0, 6.0, 7.0, 8.0, and 9.0 experimental H2ase-MFHypD FTACV data using the non-Faradaic capacitance-current model described in the Supplementary Material and both a two-sequential one-electron transfer reaction model (Reaction 1 and Reaction 2, *n*_*1*_ = *n*_2_ = 1) and the consecutive two-electron transfer model (Reaction 3, *n*_*3*_ = 2) to account for the Faradaic current. As previously (Adamson et al., 2017b), an objective function was used to quantify the distance between the total experimental current and the simulated data, denoted as $\ell_{fq}\left(x\right)$. Table 1 summarizes the best fit reaction model parameter values obtained from using the two-sequential one-electron transfer model to simulate each of three experiments at pH 4.0, 5.0, 6.0, 7.0, 8.0, and 9.0; the equivalent values for the concerted two-electron reaction model are reported in Supplementary Table S3 and the best fit non-Faradaic capacitance-current parameter values are reported in Supplementary Table S4.

**TABLE 1:** Best fit parameter values extracted when the two-sequential one-electron transfer model (*n*_*1*_ = *n*_2_ = 1) is used to simulate three repeated *ν* = 22.4 mV s^−1^, *f* = 8.96 Hz, Δ*E* = 150 mV H2ase-MFHypD FTACV experiments (Exp. 1, 2, and 3) at each of the pH values 4.0, 5.0, 6.0, 7.0, 8.0, and 9.0.

pH	Exp.	$k_{\mathrm{app}1}^{0}$ ^a^/s^−1^	$k_{\mathrm{app}2}^{0}$ ^a^/s^−1^	$E_{\mathrm{app}1}^{0}$/mV vs*.* SHE	$E_{\mathrm{app}2}^{0}$/mV vs. SHE	η ^b^/rad	Γ/pmol cm^−2^	$\ell_{\mathrm{fq}}\left(x\right)$/10^4^
4.0	1	4,000	4,000	−95.9	−95.2	2.13e-3	3.46	1.656
	2	2034	4,000	−96.9	−93.8	−2.85e-3	3.40	1.619
	3	1791	4,000	−98.5	−92.3	1.03e-3	3.32	1.635
5.0	1	2,939	4,000	−156	−149	2.26e-2	3.53	1.666
	2	2,175	4,000	−155	−149	1.81e-3	3.57	1.594
	3	4,000	4,000	−154	−150	−6.26e-3	3.55	1.607
6.0	1	4,000	4,000	−215	−206	3.64e-2	3.51	2.433
	2	1888	4,000	−213	−207	2.69e-2	3.56	1.684
	3	4,000	4,000	−214	−206	1.21e-2	3.49	1.679
7.0	1	4,000	4,000	−273	−250	6.56e-2	4.31	1.800
	2	4,000	797.8	−280	−243	1.05e-1	4.18	1.692
	3	4,000	4,000	−272	−250	5.84e-2	4.27	1.674
8.0	1	4,000	4,000	−314	−289	8.20e-2	4.02	1.800
	2	4,000	4,000	−314	−288	7.77e-2	3.97	1.632
	3	4,000	4,000	−313	−289	7.24e-2	3.97	1.642
9.0	1	4,000	4,000	−348	−326	5.15e-2	4.29	1.783
	2	4,000	4,000	−348	−326	3.84e-2	4.25	1.656
	3	4,000	4,000	−348	−326	3.24e-2	4.22	1.650

a The rate constants are reported to highlight that the reaction appears to be under equilibrium conditions across all experiments, these parameter values are therefore not well defined as the model is converging to the Nernstian limit.

b The phase was fitted about 2π in our previous work, and about 0 in this study. Therefore, although tabulated values may appear different, they are in accord.

The rate constants^a^, *k* ^0^_app1_ and *k* ^0^_app2_, and the objective function ($\ell_{fq}\left(x\right)$) are reported to 4 S.F, all other parameters are reported to 3 S.F. Phase shift^b^, η, and electrode coverage, Γ, are experiment dependent parameters rather than electron-transfer reaction parameters but affect the resulting Faradaic signal.

**FIGURE 2:**
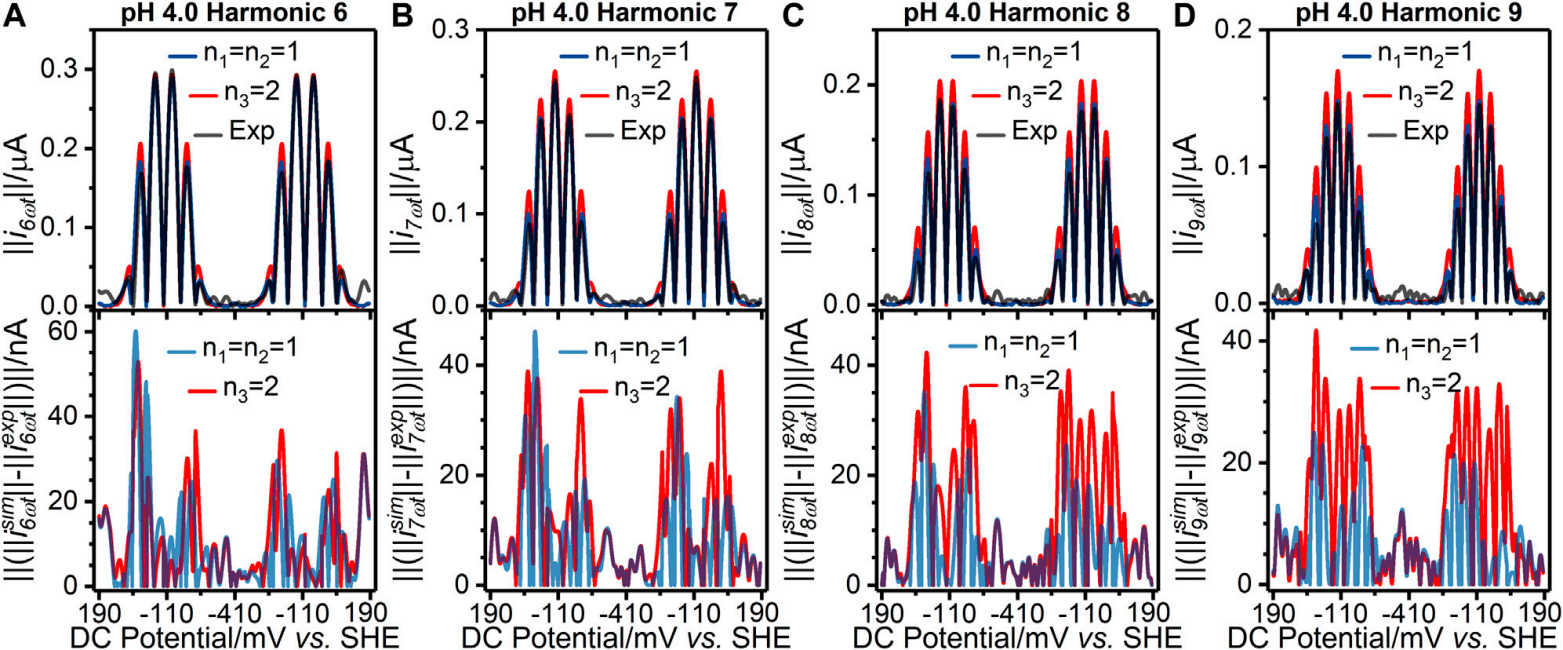
[**(A–D)** top panels] Current-potential plots of harmonics six to nine of (grey line) pH 4.0 experiment 3 (“Exp”) data and best fit simulated data using (red line) a concerted two-electron (*n*_*3*_ = 2) electron transfer model and (blue line) a stepwise two-sequential one-electron transfer reaction model (*n*_*1*_ = *n*_2_ = 1). (Bottom panels) Residual current-potential plots corresponding to the panel above. The parameter values used to generate the simulations are reported in Table 1, Supplementary Tables S3, S4.

**FIGURE 3:**
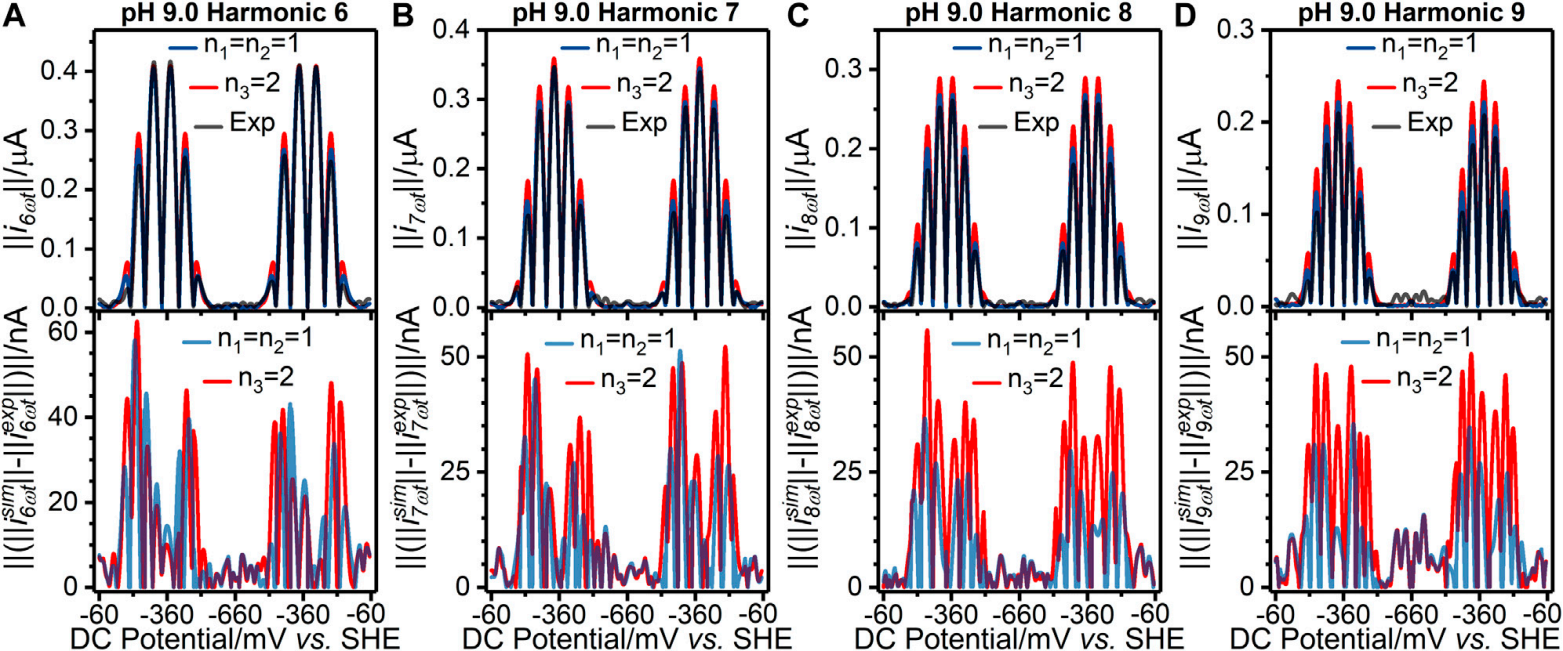
[**(A–D)** top panels] Current-potential plots of harmonics six to nine of (grey line) pH 9.0 experiment 3 (“Exp”) data and best fit simulated data using (red line) a concerted two-electron (*n*_*3*_ = 2) electron transfer model and (blue line) a stepwise two-sequential one-electron transfer reaction model (*n*_*1*_ = *n*_2_ = 1). (Bottom panels) Residual current-potential plots corresponding to the panel above. The parameter values used to generate the simulations are reported in Table 1, Supplementary Tables S3, S4.

As shown in Supplementary Tables S1, S2, the values of the objective function for the best fits from the two-sequential one-electron transfer model (*n*_*1*_ = *n*_2_ = 1) are consistently lower than those for the concerted two-electron transfer model (*n*_*3*_ = 2) across the entire pH range 4.0–9.0. The better fit of the *n*_*1*_ = *n*_2_ = 1 model can also be visualized *via* the residuals plotted in Figure 2 and Figure 3 for the pH 4.0, and 9.0 data, respectively; plots of the pH 5.0, 6.0, 7.0, and 8.0 data are shown in Supplementary Figure S8. The better fit of the *n*_*1*_ = *n*_2_ = 1 electron transfer model vs. the *n*_*3*_ = 2 electron reaction model in itself gives some confidence that this is the more likely of the two to be a correct reflection of physical reality, although it is not conclusive evidence since the better fitting model contains more free parameters (six vs. four). However, further strong evidence in favor of the correctness of the two-sequential one-electron transfer model can be seen by considering the higher harmonics of the experimental data as shown in Figures 2, 3, and Supplementart Figure S8. The fact that at least nine well defined harmonics are accessible for the reduction of surface confined H2ase-MFHypD is remarkable. This provides compelling evidence that the reduction of surface confined H2ase-MFHypD is an extremely fast process with both electron transfer and coupled protonation reactions being reversible or very close to reversible on the time scale of even the ninth harmonic of the AC voltammetric experiment. Thus, the data is strongly characteristic of a kinetic parameter approaching the reversible limit of Nernstian kinetics. This is consistent with the “best-fit” kinetic regime of the two-sequential one-electron transfer model (*n*_*1*_ = *n*_2_ = 1); as detailed below, all the electron transfer rates fall into the reversible regime (Table 1). Conversely, the concerted two-electron transfer model (*n*_*3*_ = 2) yields best-fit electron transfer ($k_{app3}^{0}$) values in the range 120–280 s^−1^, indicating the kinetic parameter is being optimized to a physically unrealistic value, consistent with model mis-specification (Supplementary Table S3).

**FIGURE 4:**
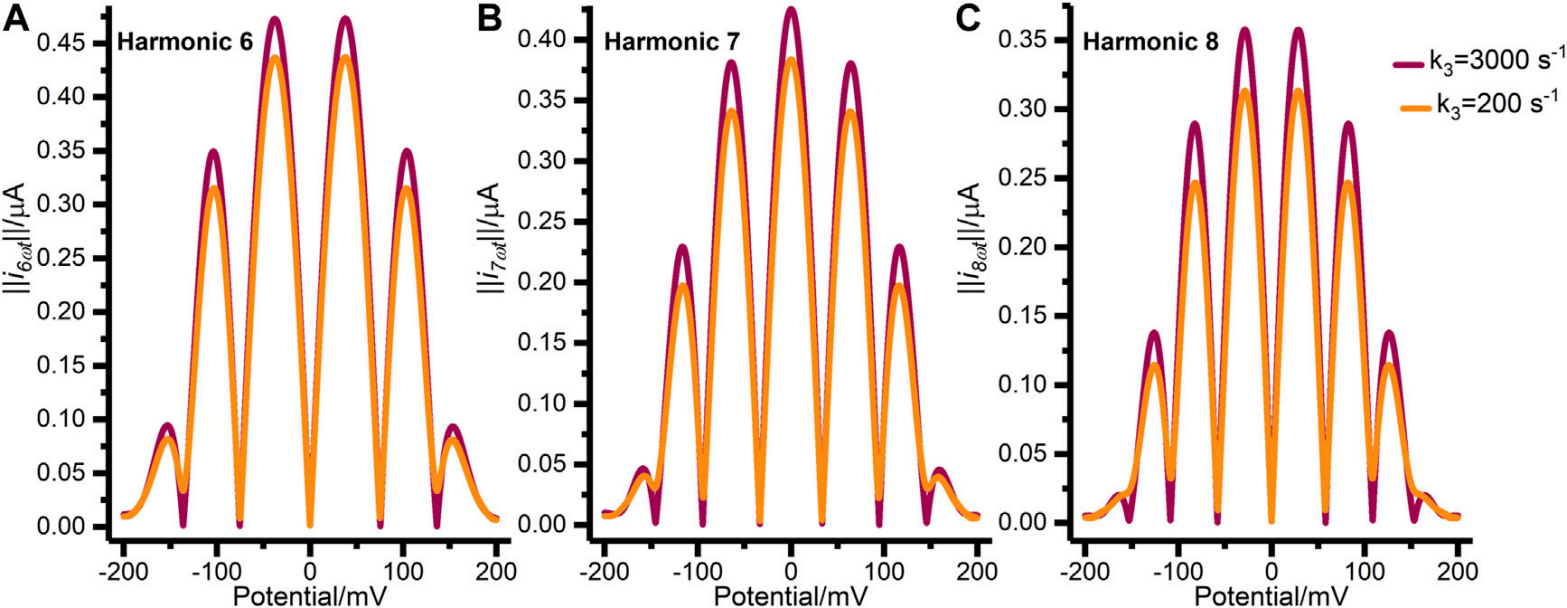
Synthetic FTACV data showing **(A)** harmonic 6, **(B)** harmonic 7, and **(C)** harmonic 8 generated by the concerted two-electron transfer model with ${\text{k}}_{3}=3,000$ s^−1^ (purple) and ${\text{k}}_{3}=200$ s^−1^ (orange). The other parameters used to generate these harmonics were: $E_{\text{start}}=200$ mV, $E_{\text{reverse}}=-200$ mV, f=9.0 Hz, ΔE=150 mV, η=0.0 rad s^−1^, ζ=1.0, $\Gamma_{0}=3.5$ pmol, S=0.03 cm^2^, v=−22.4 mVs^−1^, T=298 k, ${\text{α}}_{3}=0.5$, and uncompensated resistance and capacitance were set to zero.

Figure 4 further illustrates the mis-specification of the concerted two-electron transfer model (*n*_*3*_ = 2) by comparing data generated with a $k_{app3}^{0}$ value of 200 s^−1^ (similar to the best-fit values in Supplementary Table S3) to that generated with $k_{app3}^{0}$ value of 3,000 s^−1^ (this generates voltammetry approaching that predicted for the reversible Nernstian regime). A clear distinction between the reversible ($k_{app3}^{0}$ = 3,000 s^−1^) and quasi-reversible regimes ($k_{app3}^{0}$ = 200 s^−1^) is that in the former case the higher order AC harmonic signals for reversible electron transfer are characterized by well separated peaks and currents that drop to zero between each peak, whereas these features are lacking in the latter case, with loss in resolution of the outermost peaks being particularly evident (Figure 4). The relatively low values of the recovered “best-fit” $k_{app3}^{0}$ kinetic parameters in Supplementary Table S3 therefore give strong evidence that the concerted two-electron transfer model is mis-specified for analyzing H2ase-MFHypD data as it fails to capture key features in the high harmonic current across the range pH 4.0–9.0, as summarized in Supplementary Figure S10.

The best fit non-Faradaic capacitance-current parameters values give good estimates of background capacitance, as illustrated by Supplementary Figure S9. The polynomial parameters reported are not expected to be identical to our previous work as the model has been improved to better describe the underlying capacitance differences between the oxidative and reductive DC sweep directions (see Supplementary Material). However, the best fit values are of a comparable magnitude and as expected no significant trends are observed as a function of pH (Supplementary Table S4). In our previous work, the analysis of electrochemical impedance spectroscopy data collected at pH 6.0 was used to generate an estimate of the uncompensated resistance, $R_{\text{u}}$ ≈ 27 *Ω* (Adamson et al., 2017b), and this single value was used in all the modelling. In the current study, $R_{\text{u}}$ was fitted for every experiment and these values can be seen to vary between from 9 to 120 *Ω* (Supplementary Table S4), sitting well within the expected range. Indeed, given that the fitting method is insensitive to Ohmic IR drop within this range of uncompensated resistance (Supplementary Figure S16), no significance can be ascribed to fluctuations in these values.

Within the three experimental measurements made at the same pH, the best fit *n*_*1*_ = *n*_2_ = 1 model parameter values are generally self-consistent. Results for $E_{\text{app}1}^{0}$ and $E_{\text{app}2}^{0}$ are particularly consistent as a function of pH, and across all pH values $E_{app1}^{0}<E_{app2}^{0}$ (more in-depth analysis of the impact of pH follows later). The calculated coverage of H2ase-MFHypD on the electrode surface (Γ) is well within the sensible expected experimental range, varying between ∼3.3-4.3 pmol, as is consistent with a monolayer coverage of protein (Adamson et al., 2017b). As expected, the $E_{\text{app}1}^{0}$, $E_{\text{app}2}^{0}$ and Γ “best fit” *n*_*1*_ = *n*_2_ = 1 model parameter values for the pH 6.0 dataset are consistent with those from our previous work (Adamson et al., 2017b). Furthermore, the range covered by $E_{\text{app}1}^{0}$ and $E_{\text{app}2}^{0}$ (∼250 mV, from −95.2 to −348 mV) is consistent with previously observed mid-point potentials of disulfide bonds (∼300 mV) (Chivers et al., 1997; Chobot et al., 2007; Bewley et al., 2015).

**FIGURE 5:**
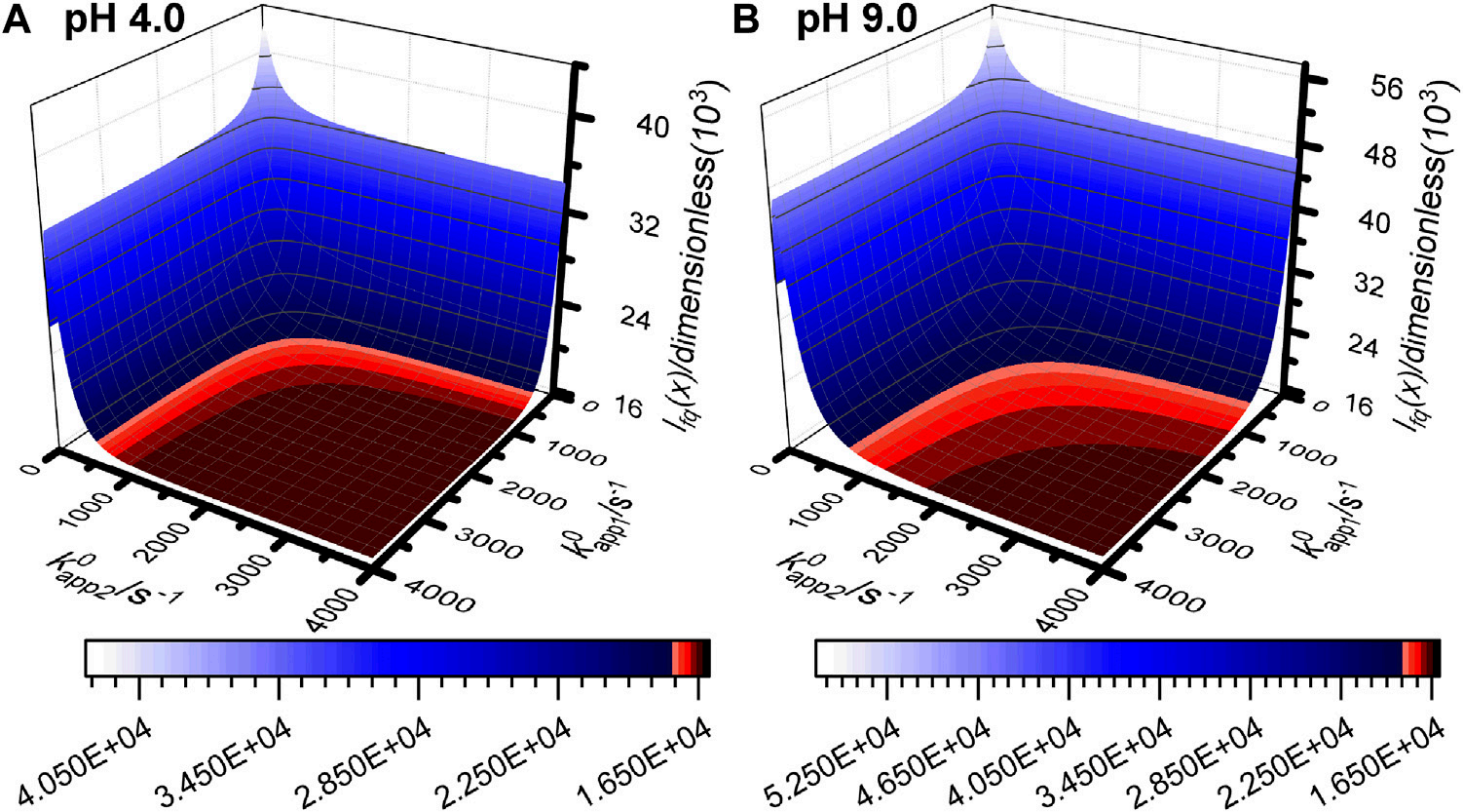
Objective function surface for **(A)** pH 4.0 experiment 3 and **(B)** pH 9.0 experiment 3 calculated using simulated data from the *n*_*1*_ = *n*_2_ = 1 reaction model with all input parameters set to the best fit values from Table 1 and Supplementary Table S4 except $k_{\text{app}1}^{0}$ and $k_{\text{app}2}^{0}$ which are varied between 0 s^−1^ and 4,000 s^−1^ and sampled every 20 s^−1^.

In our earlier work, we demonstrated that it was not possible to determine the electron transfer rate constants at pH 6.0 because the reaction reached equilibrium under the experimental conditions. This explains why experiment 1 and 3 of the pH 6.0 dataset return $k_{app1}^{0}$ and $k_{app2}^{0}$ values at the upper boundary limits that were set for these parameters (4,000 s^−1^, Supplementary Figure S7). As shown in Table 1, these rate constant boundary limits are also returned for both electron transfer processes for at least one experimental run at each pH, suggesting that across the pH range 4.0–9.0 the $k_{app1}^{0}$ and $k_{app2}^{0}$ values may be too fast to be determined using FTACV data recorded at a frequency of 8.96 Hz. This was further investigated as shown in Figure 5; using the *n*_*1*_ = *n*_2_ = 1 model to simulate pH 4.0 experiment 3 and pH 9.0 experiment 3, the $k_{\text{app}1}^{0}$ and $k_{\text{app}2}^{0}$ values were varied between 0 s^−1^ and 4,000 s^−1^ but all other model input parameters were held at their best fit values. From this analysis we see that the “quality of fit”, as determined by the objective function, is substantially unchanged above a lower rate limit of ∼2000 s^−1^, suggesting that all the experiments were conducted at a sufficiently slow timescale that the rapid biological redox process of the H2ase-MFHypD disulfide bond are under thermodynamic control.

We have not modelled the impact of either kinetic or thermodynamic dispersion when fitting the data in this work (Figures 2, 3, and Supplementary Figure S8). By definition, kinetic dispersion is not possible for a fully reversible electron transfer process that is fully described by the Nernst thermodynamic relationship. Furthermore, the experimental data shows none of the hallmarks of significant thermodynamic dispersion — a broadening of the harmonics, and substantial reduction in current amplitude of the higher harmonics relative to the non-dispersed case (Lloyd-Laney et al., 2021a). Since the harmonic simulations generated without dispersion in Figures 2, 3, and Supplementary Figure S8 provide such an excellent fit to the experimental harmonics, it is concluded that the contribution of thermodynamic dispersion is negligible.

### *E*_app_ Versus pH

**FIGURE 6:**
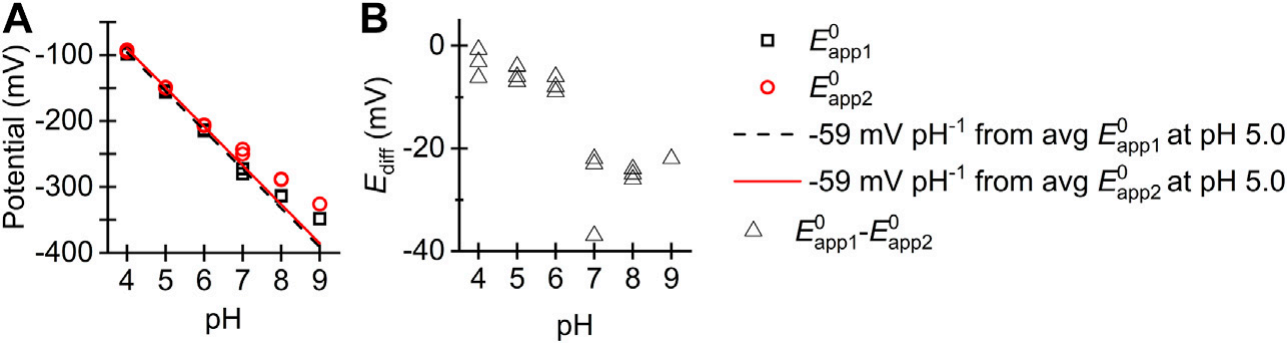
**(A)** Plot of (black square) $E_{app1}^{0}$ and (red circle) $E_{app2}^{0}$ values from Table 1 vs. pH. Overlaid are −59 mV per pH lines extrapolated from the average point values at pH 5.0 for (black dashed line) $E_{app1}^{0}$ and (red solid line) $E_{app2}^{0}$. **(B)** Plot of difference between best fit $E_{app1}^{0}$ and $E_{app2}^{0}$ values as a function of pH.

**FIGURE 7:**
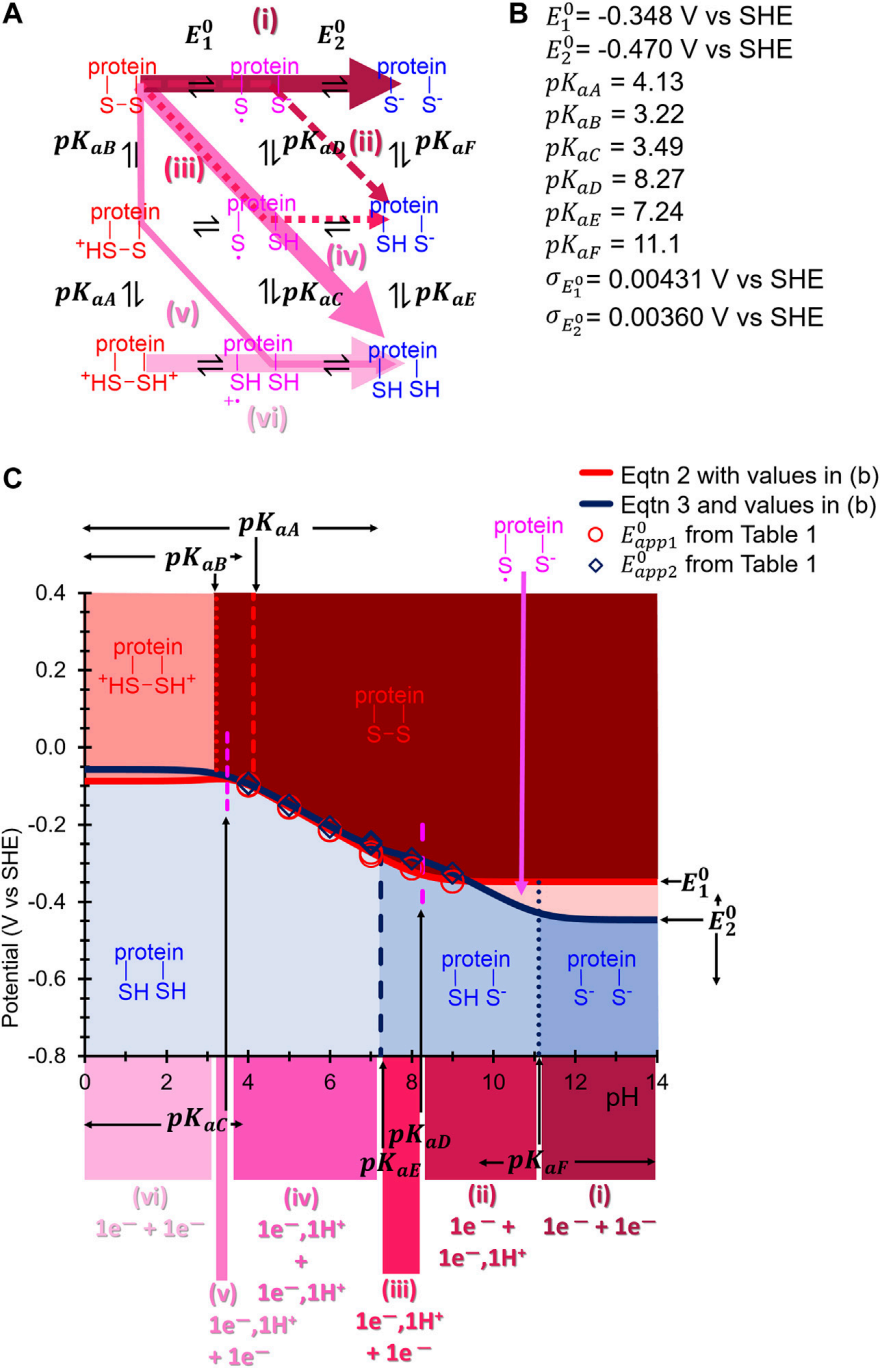
**(A)** Square scheme from Figure 1B updated to show the appropriate pKa and *E* ^0^ symbols associated with each protonation and electron-transfer reaction, respectively, of H2ase-MFHypD. Coloured arrows indicate the suggested reaction path taken through the scheme at different pH values. **(B)** Best fit inferred $pK_{a}$ and $E_{1}^{0}$, $E_{2}^{0}$ (potential values at the most alkaline pH) are calculated as described in the text. **(C)** $E_{app1}^{0}$ and $E_{app2}^{0}$ vs. pH data from Table 1 plotted along with predicted $E_{1}^{0}$, $E_{2}^{0}$ and $pK_{a}$ values shown in **(B)**. The predicted protein state is indicated by background colour and associated label within the area of the plot, and the predicted square scheme path is indicated by regions of colour underneath the *x*-axis which correspond to the arrow colours in **(A)**. The MCMC calculated parameter distribution range is shown about each parameter name in **(C)** where the distribution is wide enough to allow it to be shown in this manner.

Figure 6 shows how the $E_{\text{app}1}^{0}$ and $E_{\text{app}2}^{0}$ best-fit values for the two-sequential one-electron (*n*_*1*_ = *n*_2_ = 1) reaction model from Table 1 change as a function of pH. We observe “crossed” potentials over the entire pH range (i.e., $E_{\text{app}2}^{0}>E_{\text{app}1}^{0}$), although the potential difference notably changes, with the $E_{\text{app}1}^{0}$ and $E_{\text{app}2}^{0}$ values being within 10 mV of one another at pH 4.0, 5.0, and 6.0, then $E_{\text{app}2}^{0}$ exceeding $E_{\text{app}1}^{0}$ by about 25 mV at pH 7.0, 8.0, and 9.0. To probe this further we have used the infrastructure of the “PINTS” Python library (Clerx et al., 2019) to fit the pH vs. *E*_app_ values as inferred above (Table 1) to Eqs. 2, 3 taken from the literature (Hirst, 2006). From this approach we have inferred best fit point estimates for the characteristic $pK_{a}$ values of the square scheme in Figure 1 (note that, with reference to Figure 1, $pK_{a1}$ is equivalent to $pK_{aD}$ and $pK_{a2}$ is equivalent to $pK_{aE}$), along with the true reversible potentials, $E_{1}^{0}$ and $E_{2}^{0}$, and the standard deviation of the noise on the reversible potentials $\sigma_{E_{1}^{0}}$ and $\sigma_{E_{2}^{0}}$. Twenty optimizations were performed to maximize a Gaussian log likelihood with best fit parameters reported in Figure 7B. Additionally, parameter posterior distributions were sampled using an Adaptive Metropolis Markov Chain Monte Carlo (MCMC) method from the “PINTS” Python library, with the range of the inferred posterior distributions shown in Figure 7C. The full details of the inference approach used, along with figures detailing the posterior distributions can be found in the Supplementary Material.

$$E_{app1}^{0}=E_{1}^{0}-\frac{RT}{F}\ln\left[\left(1+\frac{\left[H^{+}\right]}{K_{aB}}+\frac{{\left[H^{+}\right]}^{2}}{K_{aA}K_{aB}}\right)÷\left(1+\frac{\left[H^{+}\right]}{K_{aD}}+\frac{{\left[H^{+}\right]}^{2}}{K_{aC}K_{aD}}\right)\right]$$ (2)

$$E_{app2}^{0}=E_{2}^{0}-\frac{RT}{F}\ln\left[\left(1+\frac{\left[H^{+}\right]}{K_{aD}}+\frac{{\left[H^{+}\right]}^{2}}{K_{aC}K_{aD}}\right)÷\left(1+\frac{\left[H^{+}\right]}{K_{aF}}+\frac{{\left[H^{+}\right]}^{2}}{K_{aE}K_{aF}}\right)\right]$$ (3)

## Discussion

Our Results indicate that across the pH range 4.0–9.0 H2ase-MFHypD maintains the same two-sequential one-electron transfer redox reaction, i.e., the Faradaic current detected in our FTACV experiments is attributable to the enzyme undergoing a redox process described by Reaction 1 and Reaction 2, converting from a fully oxidized state into a fully reduced state *via* the addition of one-electron to generate an intermediate state that accepts a second electron.

In the pH regime 4.0–6.0 the apparent potentials associated with the first and second electron transfer, $E_{\text{app}1}^{0}$ and $E_{\text{app}2}^{0}$, respectively, both alter with a gradient of approximately −59 mV per pH unit (Figure 6). The rates of the two stepwise redox-state interconversion processes are reversible on the timescale of our 8.96 Hz FTACV experiment, indicating that the experiment can be interpreted using the Nernst equation. Taken together, this evidence is consistent with the notion that in the pH range 4.0–6.0 each electron transfer is associated with a proton transfer, as derived by Laviron and many others (Laviron, 1980, 1983; Hirst, 2006; Costentin et al., 2009; Weinberg et al., 2012). Thus, in this pH regime the reaction is consistent with moving diagonally through the Pourbaix diagram in Figure 1. Therefore, from pH 4.0 to 6.0 the disulfide bond redox reaction observed in our experiments is consistent with the most oxidized state being a CysS-SCys species (the deprotonated, fully oxidized “P” species in Figure 1) that accepts one-electron and one-proton to form a radical intermediate (“PH” in Figure 1), this intermediate is further reduced by one-proton and one-electron to form the CysSH HSCys reduced species (“PH_2_” in Figure 1).

**FIGURE 8:**
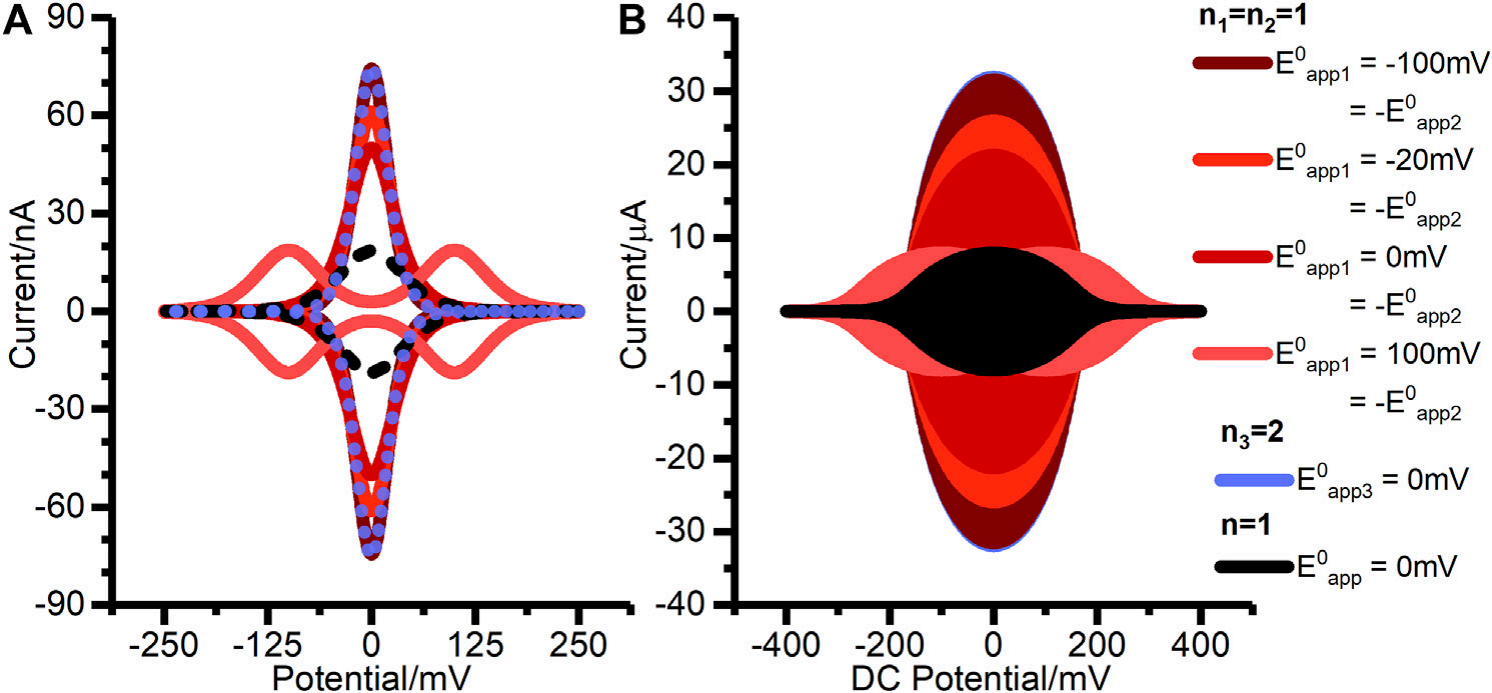
Simulated **(A)** DCV, and **(B)** FTACV outputs for two-stepwise one-electron transfer processes (*n*_*1*_ = *n*_2_ = 1) when (solid pink line) $E_{app1}^{0}$ > $E_{app2}^{0}$ (solid light red line) $E_{app1}^{0}$ = $E_{app2}^{0}$, and (solid orange and dark red lines) $E_{app2}^{0}$ > $E_{app1}^{0}$. The data is also compared to (dashed black line) a single, reversible one electron transfer (*n* = 1), and (dotted blue line) a single, reversible two-electron (*n*_*3*_ = 2) transfer. For the DCV simulations in **(A)** all electron transfer rate constants were set to 10,000 s^−1^, while for the simulations FTACV in **(B)** all electron transfer rate constants were set to 100,000 s^−1^. The other parameters used for these simulations were: η=0.0 rad s^−1^, ζ=1.0, $\Gamma_{0}=10.0$ pmol, S=0.01 cm^−2^, v=−20 mVs^−1^, T=298.15 k, all α values were set to 0.5, and uncompensated resistance and capacitance were set to zero. For **(A)** $E_{start}=250$ mV, $E_{\text{reverse}}=-250$ mV, f=0.0 Hz, and ΔE=0 mV while for **(B)** $E_{\text{start}}=400$ mV, $E_{\text{reverse}}=-400$ mV, f=9.0 Hz, and ΔE=150 mV. The *n* = 1 simulations were completed using the two-stepwise one-electron transfer model and setting the second rate constant ($k_{\text{app}2}^{0}$) to zero.

Although the net reaction is consistent with Figure 1A, we find that a concerted two-electron, two-proton mechanism is not appropriate, i.e., the classic double-headed arrow of organic chemistry does not accurately describe the reaction. A quasi-reversible concerted two-electron model is found to be mis-specified, as described in the Results. Figure 8 shows a theoretical comparison of the DCV and FTACV output from reversible concerted two-electron transfer compared to reversible two sequential one-electron transfers in the regimes of $E_{\text{app}1}^{0}$ = $E_{\text{app}2}^{0}$ and $E_{\text{app}1}^{0}$ = $E_{\text{app}2}^{0}$+40 mV ($E_{\text{app}1}^{0}$ = −20 mV = $-E_{\text{app}2}^{0}$ in Figure 8) and this analysis confirms that the two reversible reaction types will be distinguishable (the plot shows that the reaction models have converged when $E_{\text{app}1}^{0}$ = −100 mV = $-E_{\text{app}2}^{0}$, i.e., when $E_{\text{app}2}^{0}=E_{\text{app}1}^{0}+200mV$). Therefore, detectable amounts of the intermediate radical generated during the redox reaction of H2ase-MFHypD will exist at the midpoint potential defined by averaging $E_{\text{app}1}^{0}$ and $E_{\text{app}2}^{0}$ (Supplementary Figure S2). This is consistent with the observation of radical species in EPR experiments designed to probe X-ray damage in crystallography experiments on disulfide-containing proteins (Sutton et al., 2013), and also with observations of reactions between simple disulfides and one-electron reducing agents (Hoffman and Hayon, 1972).

When constructing Figure 8 we found that the magnitude of the electron transfer rate constant required to achieve model convergence in the $E_{\text{app}1}^{0}$= −100 mV = $-E_{\text{app}2}^{0}$ regime is considerably greater for FTACV than for DCV. Thus, while rate constants of 10,000 s^−1^ were used for the DCV plot in Figure 8A, equivalent values of 100,000 s^−1^ were used for the FTACV simulations. This larger rate constant is needed as model convergence is only possible when in the reversible regime. This highlights the utility of the FTACV technique in differentiating between reaction types, although it is notable that this theoretical consideration does not account for noise, resistance, dispersion, or background current contributions, all of which limit the accuracy of reaction model determination (Lee et al., 2011; Stevenson et al., 2012; Adamson et al., 2017a; Adamson et al., 2017b; Lloyd-Laney et al., 2021b). The effects of noise and background current contribution on real data are particularly well illustrated in our previous work on H2ase-MFHypD, which shows that many of the difficulties in model differentiation from DCV data are removed in FTACV analysis due to the increased ratio of Faradaic to capacitive current in FTACV.

In amino acid reference tables, cysteine residues are ascribed an average pKa value of 8.5 (Poole, 2015), suggesting that under sufficiently alkali conditions the reductive cleavage of a disulfide linkage should collapse into a two-electron, zero-proton regime, equivalent to the top line of the Pourbaix diagram in Figure 1. The increased gap between $E_{\text{app}1}^{0}$ and $E_{\text{app}2}^{0}$ at pH 7.0, 8.0, and 9.0 relative to the more acidic conditions (see Table 1 and Figure 6) is consistent with a change in the ratio of protons to electrons at pH ≥ 6.0 and when we fit the data to Eqs. 2, 3 we derive a $pK_{a}$ value of 7.3 for deprotonation of the CysSH HSCys reduced state, and a $pK_{a}$ of 8.3 for deprotonation of the CysS HSCys intermediate oxidation state species. The lack of the experimental data in the regimes of 3.5 < pH > 9.5 means that we cannot be confident in the accuracy of the remaining 4 $pK_{a}$ values detailed in Figure 7 (Supplementary Figure S14A for posterior distrubutions). However, from the ranges shown in Figure 7 (and Supplementary Figure S14A) it can be seen that the ordering of $pK_{aD}$, $pK_{aE}$, and $pK_{aF}$ is very unlikely to change, while there is some uncertainty around the ordering of $pK_{aA}$, $pK_{aB}$, and $pK_{aC}$. Consequently, even though the *E*_app_ data is sparse and exclusively at equilibrium, we can still make predictions about the likely possible paths through the square scheme within H2ase-MFHypD. This illustrates the power of our approach. In addition, it suggests that high-frequency data, where specific estimates of the kinetic parameter can be obtained, along with the possibility of determining proton transfer rates, will be an even richer source of biological information.

With this in mind, we wish to draw the reader’s attention to the incredible role that the protein matrix must play in ensuring a highly consistent redox reaction mechanism is maintained over a range of solution protonation values from 10^-4^ to 10^-9^ M. Clearly the secondary protein structure plays a vital role in mediating the protonation environment to enable Biology to precisely tune the disulfide chemistry so that the $E_{\text{app}1}^{0}$ and $E_{\text{app}2}^{0}$ values remain remarkably in concert across a very wide range. The fact that the redox chemistry of H2ase-MFHypD is consistent with the “classical” treatment of proton-coupled electron transfer *via* a square-scheme description of separated, stepwise EC processes across such a wide pH window is notable because at pH 9.0 so few protons are available. Thus, this work offers further evidence to the hypothesis that establishing stable radical one-electron reduced intermediates is a conserved feature in much of the two-electron redox chemistry seen in biology (Evans et al., 2019).

There are examples of related voltammetric behaviour outside that of biologically relevant molecules. A well-studied example is the reduction of solution soluble and surface confined polyoxometalates as a function of acid concentration. For example, the DC cyclic voltammetry for reduction of [P_2_W_18_O_62_]^6-^ in acetonitrile provides an extensive series of well separated one-electron reduction processes. On addition of sufficient acid, the processes converge into apparently multi-electron transfer processes (Prenzler et al., 1999). However, detailed voltammetry simulations based on one-electron transfer steps model the initial two reactions very well over the acid concentration range. The acid dependence and simulation details for this and related polyoxometalates have been reviewed (Bond, 2002). A fully analogous situation to the present study (Robinson et al., 2018; Robinson et al., 2019) has been demonstrated to apply for surface confined reduction of [PMo_12_O_40_]^3-^; in highly acid media it is shown to be well modelled by treatment as two closely spaced one-electron reduction steps rather than a simultaneous two-electron reduction processes. Conversely, more exotic electron-proton transfer schemes like the elegant “wedge” scenario are necessary for describing electron and proton transfer *via* an intermediate H-bond complex in some organic molecules (Clare et al., 2019).

## Data Availability

The datasets presented in this study can be found in online repositories. The names of the repository/repositories and accession number(s) can be found below: https://github.com/alisterde/HypD_low_freq_pH_analysis
