# Supplementary material for: A Voltammetric Perspective of Multi-Electron and Proton Transfer in Protein Redox Chemistry: Insights From Computational Analysis of Escherichia coli HypD Fourier Transformed Alternating Current Voltammetry
Source: Front Chem. 2021 Jun 14;9:672831. doi: 10.3389/fchem.2021.672831 (PMC8238118; doi:10.3389/fchem.2021.672831)
Supplement: Supplementary file 1 [file DataSheet1.pdf]

Supplementary Material for "A Voltammetric Perspective of Multi-Electron and Proton Transfer in Protein Redox Chemistry: Insights from Computational Analysis of Escherichia coli HypD Fourier Transformed Alternating Current Voltammetry"

Contents

1 H2ase-MFHypD DC and FTACV data at pH 6.0 S2
2 Nernst plot S3
3 H2ase-MFHypD FTACV data from pH 4.0 to pH 9.0 S4
4 Mathematical model S5
4.1 Model for two-stepwise one-electron transfers
4.1.1 Dimensionless equations
4.2 Model for one-concerted two-electron transfer
5 Computational methods S11
5.1 Forward problem
5.2 Inverse problem
5.2.1 Finding best fit parameter values for the non-Faradaic capacitance-current model
5.2.2 Finding best fit parameter values for the two different reaction models
5.3 Verification of computational methods
5.3.1 Forward problem verification
5.3.2 Inverse problem verification
6 Comparing best fit objective function values for the two models S18
7 Comparing pH 5.0, 6.0, 7.0 and 8.0 experimental data and best fit plots from both reaction models S19
8 Best fit parameter values for the one-concerted two-electron transfer model S20
9 Best fit capacitance parameter values S21
10 Misspecification of the n3 = 2 model S23
11 Verification of H2ase-MFHypD Signal S25
12 Inference and MCMC for Eapp versus pH S26
13 Insensitivity of Faradaic fitting to Uncompensated Resistance S31
14 References S32

1 H2ase-MFHypD DC and FTACV data at pH 6.0

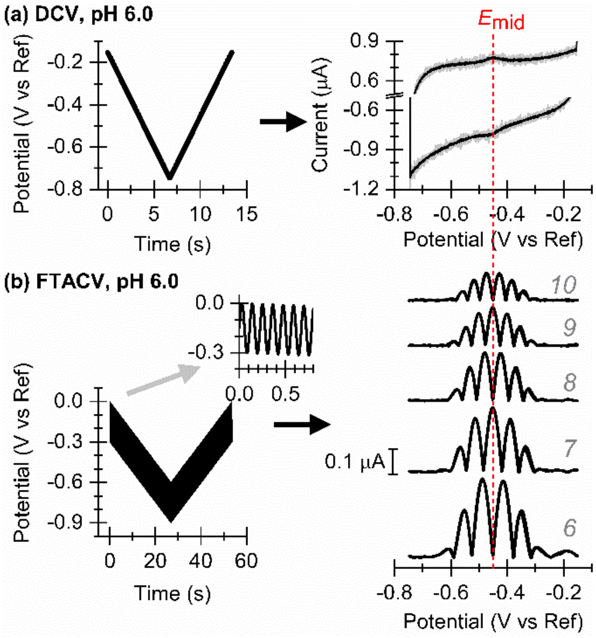

Figure S1 Comparison of pH 6.0 H2ase-MFHypD (a) DCV at a scan rate of 89 mV s^{-1} and (b) FTACV data obtained with an underlying DC scan rate of 22.4 mV s^{-1} and a sinewave perturbation of amplitude 150 mV and frequency of 8.96 Hz, where the potential-time methodologies are contrasted as well as the resulting current-potential output voltammogram of DCV and 6th-10th harmonics of FTACV (grey letters indicate harmonic number). The vertical red dotted line indicates how the reversible midpoint potential associated with the overall redox reaction (E_{mid}) is extracted from each experiment.

2 Nernst plot

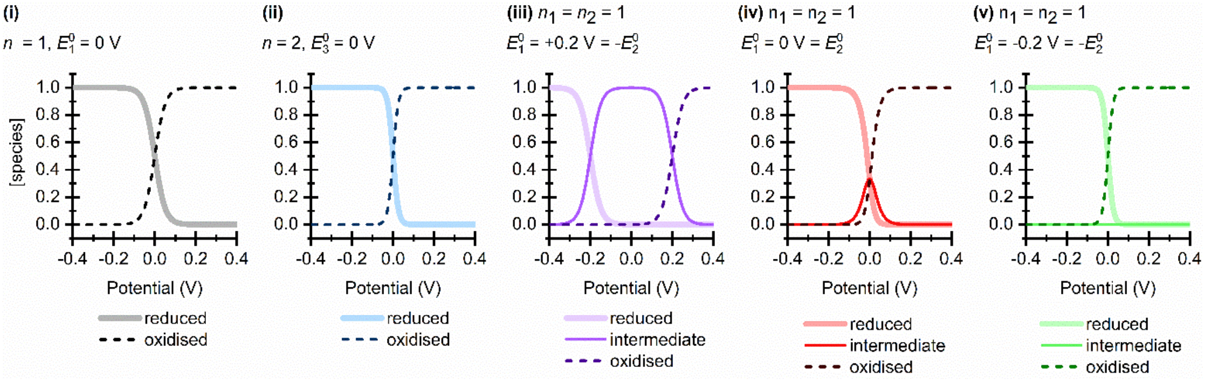

Figure S2 Plots derived from the Nernst equation showing how under equilibrium conditions the amount of an "intermediate" state generated during two-stepwise, reversible one-electron transfers depends on the difference in the reversible potentials governing each one-electron process. "Intermediate" is formed by adding one-electron ($n_1 = 1$) to the "oxidised" state (equilibrium potential = E_1^0) and it is consumed by a one-electron reduction ($n_2 = 1$) that produces the "reduced" state (equilibrium potential = E_2^0). For comparison, plots for a single one-electron "n = 1" reversible reduction process (equilibrium potential = E_1^0) and simultaneous "$n_3 = 2$" process (equilibrium potential = E_3^0) are also included, the latter is indistinguishable from the two-stepwise one-electron transfers when E_1^0 is sufficiently more negative than E_2^0.

3 H2ase-MFHypD FTACV data from pH 4.0 to pH 9.0

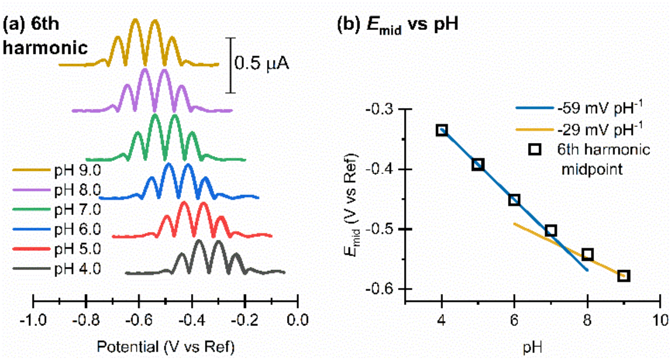

Figure S3 Stack-plot comparison of 6th harmonic H2ase-MFHypD FTACV experimental data collected previously (Adamson et al., 2017b). FTACV experiments were conducted with dc scan rate (v) = 22.4 mV s^{-1}, AC sinewave frequency (f) = 8.96 Hz, and AC sinewave amplitude (ΔE) = 150 mV. Experimental pH values are as indicated by the legend. (b) Scatter plot of the midpoint potentials extracted from experiments shown in (a) and reference lines indicating (blue) -59 mV pH^{-1} gradient extrapolated from the pH 6.0 experimental data point and (yellow) -29 mV pH^{-1} gradient extrapolated from the pH 9.0 experimental data point.

4 Mathematical model

The mathematical model used is derived from that described in our previous work on H2ase-MFHypD (Adamson et al., 2017b), with changes to the capacitive current calculations (*vide infra*). As described in the main paper, two different reaction regimes are considered, both a two stepwise one-electron transfer reaction (n_1=n_2=1), where a surface-confined intermediate oxidation state is formed, $Y_{(surf)}$, as in Reaction S1 and Reaction S2 (Reactions 1 and 2 in main paper); and a simultaneous two-electron transfer reaction (n_3=2) to convert the surface-confined oxidised species ($X_{(surf)}$) into a surface-confined reduced species ($Z_{(surf)}$), as in Reaction S3 (Reaction 3 in the main paper). In all cases, k_i^{red} is the potential-dependent reductive/forward rate constant for reaction i, and k_i^{ox} is the potential-dependent oxidative/backwards rate constant for reaction i.

$$\mathbf{X}_{(\mathrm{surf})} + \mathbf{e}^- \underset{k_1^{\mathrm{ox}}}{\overset{k_1^{\mathrm{red}}}{\rightleftharpoons}} \mathbf{Y}_{(\mathrm{surf})} \quad (E^0_{app1}, k^0_{app1}, \alpha^0_{app1})$$ Reaction S1

$$\mathbf{Y}_{(\mathrm{surf})} + e^- \underset{k_2^{\mathrm{ox}}}{\overset{k_2^{\mathrm{red}}}{\rightleftharpoons}} \mathbf{Z}_{(\mathrm{surf})} \quad (E^0_{app2}, k^0_{app2}, \alpha^0_{app2})$$ Reaction S2

$$\mathbf{X}_{(\mathrm{surf})} + 2\mathbf{e}^- \underset{k_3^{\mathrm{ox}}}{\overset{k_3^{\mathrm{red}}}{\rightleftharpoons}} \mathbf{Z}_{(\mathrm{surf})} \quad (E^0_{app3}, k^0_{app3}, \alpha^0_{app3})$$ Reaction S3

The "app" subscript is used to denote the fact that conditions cannot be achieved where protonation is absent, so that true E^0 values for non-proton coupled electron transfer processes are unknown, as are protonation equilibrium K_a values. Instead, it is assumed that protonation reactions are reversible (diffusion controlled), and modelling is therefore undertaken by combining the E^o, K_a and pH terms into an apparent "E^o_{app}" value which is defined as the reversible potential (Bond, 2002; Adamson et al., 2017b). This is possible because the processes maintain the same shape and are simply shifted to less positive potentials by the reversible chemical reactions following the electron transfer step. On this basis, the heterogeneous charge transfer rate constant k^0_{app} and charge transfer coefficient α^0_{app} are the electrode kinetic parameters relevant to E^0_{app}.

Using Butler Volmer theory (Bard and Faulkner, 2000), the forwards $k_i^{red}(t)$ and backwards $k_i^{ox}(t)$ potential dependent reaction rate constants for an electron transfer step at time t are written as:

$$k_i^{red}(t) = k^0_{app\,i}\exp\left(-\frac{\alpha_i n_i F}{RT}\left[E_r(t) - E^0_{app\,i}\right]\right),$$ Equation S1

$$k_i^{ox}(t) = k^0_{app\,i}\exp\left((1-\alpha_i)\frac{n_i F}{RT}\left[E_r(t) - E^0_{app\,i}\right]\right),$$ Equation S2

where the subscript i denotes the reaction and the superscript 0 indicates stated conditions, $k^0_{app\,i}$ is the electron transfer rate coefficient, α_i is the charge transfer coefficient, F is the Faraday constant, R is the gas constant, T is temperature in Kelvin, n_i is the number of

electrons transferred in the reaction, and $E_{\mathrm{r}}(t)$ is the real (effective) potential seen by the working electrode at time t.

$E_{\mathrm{r}}(t)$, differs from the input applied potential $E(t)$ due to uncompensated resistance R_{u} resulting in an 'ohmic drop' (IR_{u}). In electrochemistry ohmic drop is the result of the potential induced by the resistance of the electrolyte or any other interface such as surface films or connectors. $E_{\mathrm{r}}(t)$ can therefore be formulated as:

$$\boldsymbol{E_{\mathrm{r}}(t) = E(t) - R_{\mathrm{u}} I_{\mathrm{tot}}(t)}, \qquad \text{Equation S3}$$

where $I_{\mathrm{tot}}(t)$ is the total current measured and is described later.

In FTACV the input potential as a function of time, $E(t)$, is formulated as Equation S4, the same equation is also given in the main paper:

$$\boldsymbol{E(t) = E_{\mathrm{dc}}(t) + \Delta E \sin(\omega t + \eta)}\,. \qquad \text{Equation S4}$$

The first term of Equation S4, $E_{\mathrm{dc}}(t)$, describes dc contributions to the overall input potential, while the second term, $\Delta E \sin(\omega t + \eta)$, describes the ac contribution; ΔE is the amplitude of the sine wave, ω is the angular frequency ($\omega = 2\pi f$, where f is frequency in Hz), and η is the phase. Thus, DCV can be accommodated by Equation S4, as the second term will be zero.

Furthermore, $E_{\mathrm{dc}}(t)$ is formulated accordingly to accommodate cyclic voltammetry:

$$\boldsymbol{E_{\mathrm{dc}}(t) = \begin{cases} E_{\mathrm{start}} + vt\,, & \text{for } 0 \le t < t_{\mathrm{r}} \\ E_{\mathrm{reverse}} - v(t - t_{\mathrm{r}})\,, & \text{for } t_{\mathrm{r}} \le t \end{cases}} \qquad \text{Equation S5}$$

$$\boldsymbol{t_{\mathrm{r}} = \frac{E_{\mathrm{reverse}} - E_{\mathrm{start}}}{v}}\,, \qquad \text{Equation S6}$$

where v is the dc scan rate (i.e. rate of change of the linear dc ramp), E_{start} is the initial applied potential, and E_{reverse} is the potential at which the direction of potential change reverses.

4.1 Model for two-stepwise one-electron transfers

To model Reaction S1 and Reaction S2, we let the proportion of surface-confined species in state X be θ_X, the proportion of surface-confined species in state Y be θ_Y, and the proportion of surface-confined species in state Z be θ_Z, with initial conditions $\theta_\mathrm{X}(0) = 1$, $\theta_\mathrm{Y}(0) = 0$, and $\theta_\mathrm{Z}(0) = 0$. The ordinary differential equations governing their behaviour are given by:

$$\frac{\mathbf{d}\boldsymbol{\theta}_\mathrm{X}}{\mathbf{d}\boldsymbol{t}} = \boldsymbol{k}_1^{\mathrm{ox}}(\mathbf{1} - \boldsymbol{\theta}_\mathrm{X} - \boldsymbol{\theta}_\mathrm{Z}) - \boldsymbol{k}_1^{\mathrm{red}}\boldsymbol{\theta}_\mathrm{X}\,, \qquad \text{Equation S7}$$

$$\frac{\mathbf{d}\boldsymbol{\theta}_\mathrm{Z}}{\mathbf{d}\boldsymbol{t}} = \boldsymbol{k}_2^{\mathrm{red}}(\mathbf{1} - \boldsymbol{\theta}_\mathrm{X} - \boldsymbol{\theta}_\mathrm{Z}) - \boldsymbol{k}_2^{\mathrm{ox}}\boldsymbol{\theta}_\mathrm{Z}\,. \qquad \text{Equation S8}$$

Note the substitution $\theta_\mathrm{Y} = 1 - \theta_\mathrm{X} - \theta_\mathrm{Z}$ has been used and $\frac{\mathrm{d}\theta_\mathrm{Y}}{\mathrm{d}t}$ has been omitted as $\frac{\mathrm{d}\theta_\mathrm{Y}}{\mathrm{d}t} = -\frac{\mathrm{d}\theta_\mathrm{X}}{\mathrm{d}t} - \frac{\mathrm{d}\theta_\mathrm{Z}}{\mathrm{d}t}$, it is therefore not independent and the system may be fully described without it.

There are two contributions to the total current, I_tot, a capacitive term (I_c) and a Faradaic term (I_f), yielding:

$$\boldsymbol{I}_\mathrm{tot} = \boldsymbol{S}\boldsymbol{I}_\mathrm{c} + \boldsymbol{I}_\mathrm{f}\,, \qquad \text{Equation S9}$$

where I_c is defined in units of amps per unit area, as is standard, and is therefore scaled by electrode area S (assumed to equal the geometric area, 0.03 cm^2). The Faradaic term is due to the electrons transferred between the enzyme and the electrode, and is therefore formulated:

$$\boldsymbol{I}_\mathrm{f} = \boldsymbol{F}\boldsymbol{S}\boldsymbol{\Gamma}\left(\frac{\mathbf{d}\boldsymbol{\theta}_\mathrm{X}}{\mathbf{d}\boldsymbol{t}} - \frac{\mathbf{d}\boldsymbol{\theta}_\mathrm{Z}}{\mathbf{d}\boldsymbol{t}}\right), \qquad \text{Equation S10}$$

where Γ is the surface coverage of enzyme per unit area. The electrode used in the experiment lacks a reliable model for the capacitive term, therefore the following third order polynomial in E_r is used:

$$\boldsymbol{I}_\mathrm{c} = (\boldsymbol{C}_\mathrm{dl} + \boldsymbol{C}_\mathrm{dl1}\boldsymbol{E}_\mathrm{r}(\boldsymbol{t}) + \boldsymbol{C}_\mathrm{dl2}\boldsymbol{E}_\mathrm{r}^2(\boldsymbol{t}) + \boldsymbol{C}_\mathrm{dl3}\boldsymbol{E}_\mathrm{r}^3(\boldsymbol{t}))\frac{\mathbf{d}\boldsymbol{E}_\mathrm{r}}{\mathbf{d}\boldsymbol{t}}\,, \qquad \text{Equation S11}$$

where C_dl, C_dl1, C_dl2, and C_dl3 are best fit constants. In this work, different constantsC_dl, C_dl1, C_dl2, and C_dl3 are defined for the regions of the experiment either side of the E_reverse value, this is described by Equation S12 to Equation S15.

$$\boldsymbol{C}_\mathrm{dl} = \begin{cases}\boldsymbol{C}_\mathrm{vdl}, & \mathbf{for}\ \mathbf{0} \le \boldsymbol{t} < \boldsymbol{t}_\mathrm{r} \\ \boldsymbol{C}_{-\mathrm{vdl}}, & \mathbf{for}\ \boldsymbol{t}_\mathrm{r} \le \boldsymbol{t}\end{cases} \qquad \text{Equation S12}$$

$$\boldsymbol{C}_\mathrm{dl1} = \begin{cases}\boldsymbol{C}_\mathrm{vdl1}, & \mathbf{for}\ \mathbf{0} \le \boldsymbol{t} < \boldsymbol{t}_\mathrm{r} \\ \boldsymbol{C}_{-\mathrm{vdl1}}, & \mathbf{for}\ \boldsymbol{t}_\mathrm{r} \le \boldsymbol{t}\end{cases} \qquad \text{Equation S13}$$

$$\boldsymbol{C}_{\mathrm{dl2}}=\begin{cases}\boldsymbol{C}_{\mathrm{vdl2}}, & \textbf{for } \mathbf{0}\leq \boldsymbol{t}<\boldsymbol{t}_{\mathrm{r}}\\ \boldsymbol{C}_{-\mathrm{vdl2}}, & \textbf{for } \boldsymbol{t}_{\mathrm{r}}\leq \boldsymbol{t}\end{cases}$$ Equation S14

$$\boldsymbol{C}_{\mathrm{dl3}}=\begin{cases}\boldsymbol{C}_{\mathrm{vdl3}}, & \textbf{for } \mathbf{0}\leq \boldsymbol{t}<\boldsymbol{t}_{\mathrm{r}}\\ \boldsymbol{C}_{-\mathrm{vdl3}}. & \textbf{for } \boldsymbol{t}_{\mathrm{r}}\leq \boldsymbol{t}\end{cases}$$ Equation S15

The new capacitance model provides a superior fit to the background capacitative current when compared to the model used in our previous work which assumed the background current was independent of scan direction (Adamson et al., 2017b). This is attributed to two reasons: firstly, the new capacitance model incorporates more parameters, therefore it has more degrees of freedom. Secondly, the new model accounts for the physical reality that in our system we would not expect capacitance to be independent of scan direction. If we consider the negative DC current scan direction, ideally complete reduction of the surface confined species will have been achieved by the end of this voltage sweep, resulting in an electrode surface modified by the presence of the reduced species. The presence of a reduced surface-confined species can provide a different contribution to the background current to that encountered with surface-confined species prior to reduction. This potential dependent surface state will lead to hysteresis in the background current, with the profile varying with scan direction. The use of a different models to describe the background when sweeping the potential in the negative and positive directions accommodates this hysteresis.

4.1.1 Dimensionless equations

To aid computation, Equation S7 to Equation S9 were represented in a dimensionless form by substituting the dimensionless variables $i = I_{\mathrm{tot}}/I_0$, $\tau = t/T_0$, and $\epsilon = E/E_0$ for all potentials and setting:

$$\boldsymbol{E}_{\mathbf{0}}=\frac{\boldsymbol{RT}}{\boldsymbol{F}},\qquad \boldsymbol{T}_{\mathbf{0}}=\frac{\boldsymbol{E}_{\mathbf{0}}}{\boldsymbol{v}},\qquad \boldsymbol{I}_{\mathbf{0}}=\frac{\boldsymbol{FS\Gamma}_{\mathbf{0}}}{\boldsymbol{T}_{\mathbf{0}}}.$$ Equation S16

Γ_0 is an approximation of electrode coverage with units of moles per unit area. The time dependent variables θ_{X} and θ_{Z} are already dimensionless and therefore remain. For Equation S7 this gives:

$$\frac{\mathrm{d}\boldsymbol{\theta}_{\mathrm{X}}}{\mathrm{d}\boldsymbol{\tau}}=\boldsymbol{\kappa}_1^{\mathbf{0}}\left((\mathbf{1}-\boldsymbol{\theta}_{\mathrm{X}}-\boldsymbol{\theta}_{\mathrm{Z}})\boldsymbol{e}^{(1-\alpha_1)(\epsilon_{\mathrm{r}}-\epsilon_1^0)}-\boldsymbol{\theta}_{\mathrm{X}}\boldsymbol{e}^{-\alpha_1(\epsilon_{\mathrm{r}}-\epsilon_1^0)}\right),$$ Equation S17

where $\kappa_1^0 = k_{\mathrm{app1}}^0 T_0$ and is the dimensionless reaction kinetics parameter. For Equation S8 it gives:

$$\frac{\mathrm{d}\boldsymbol{\theta}_{\mathrm{Z}}}{\mathrm{d}\boldsymbol{\tau}}=\boldsymbol{\kappa}_2^{\mathbf{0}}\left((\mathbf{1}-\boldsymbol{\theta}_{\mathrm{X}}-\boldsymbol{\theta}_{\mathrm{Z}})\boldsymbol{e}^{-\alpha_2(\epsilon_{\mathrm{r}}-\epsilon_2^0)}-\boldsymbol{\theta}_{\mathrm{Z}}\boldsymbol{e}^{(1-\alpha_2)(\epsilon_{\mathrm{r}}-\epsilon_2^0)}\right),$$ Equation S18

where the dimensionless reaction kinetics parameter is $\kappa_2^0 = k_{\mathrm{app2}}^0 T_0$. While for Equation S9 it yields:

$$\boldsymbol{i} = (\boldsymbol{\gamma} + \boldsymbol{\gamma_1}\boldsymbol{\epsilon}_{\mathrm{r}} + \boldsymbol{\gamma_2}\boldsymbol{\epsilon}_{\mathrm{r}}^2 + \boldsymbol{\gamma_3}\boldsymbol{\epsilon}_{\mathrm{r}}^3)\frac{\mathbf{d}\boldsymbol{\epsilon}_{\mathrm{r}}}{\mathbf{d}\boldsymbol{\tau}} + \boldsymbol{\zeta}\left(\frac{\mathbf{d}\boldsymbol{\theta}_{\mathrm{X}}}{\mathbf{d}\boldsymbol{\tau}} - \frac{\mathbf{d}\boldsymbol{\theta}_{\mathrm{Z}}}{\mathbf{d}\boldsymbol{\tau}}\right), \quad \text{Equation S19}$$

where the dimensionless electrode coverage $\zeta = FS\Gamma/T_0I_0 = \Gamma/\Gamma_0$ (ζ is introduced to adjust the surface coverage Γ); and the dimensionless double layer capacitance parameters are:

$$\boldsymbol{\gamma} = \begin{cases} \boldsymbol{C}_{\mathrm{vdl}}\boldsymbol{E}_0\boldsymbol{S}/\boldsymbol{T}_0\boldsymbol{I}_0, & \mathbf{for}\ \mathbf{0} \le \boldsymbol{\tau} < \boldsymbol{\tau}_{\mathrm{r}} \\ \boldsymbol{C}_{-\mathrm{vdl}}\boldsymbol{E}_0\boldsymbol{S}/\boldsymbol{T}_0\boldsymbol{I}_0, & \mathbf{for}\ \boldsymbol{\tau}_{\mathrm{r}} \le \boldsymbol{\tau} \end{cases} \quad \text{Equation S20}$$

$$\boldsymbol{\gamma_1} = \begin{cases} \boldsymbol{C}_{\mathrm{vdl1}}(\boldsymbol{E}_0)^2\boldsymbol{S}/\boldsymbol{T}_0\boldsymbol{I}_0, & \mathbf{for}\ \mathbf{0} \le \boldsymbol{\tau} < \boldsymbol{\tau}_{\mathrm{r}} \\ \boldsymbol{C}_{-\mathrm{vdl1}}(\boldsymbol{E}_0)^2\boldsymbol{S}/\boldsymbol{T}_0\boldsymbol{I}_0, & \mathbf{for}\ \boldsymbol{\tau}_{\mathrm{r}} \le \boldsymbol{\tau} \end{cases} \quad \text{Equation S21}$$

$$\boldsymbol{\gamma_2} = \begin{cases} \boldsymbol{C}_{\mathrm{vdl2}}(\boldsymbol{E}_0)^3\boldsymbol{S}/\boldsymbol{T}_0\boldsymbol{I}_0, & \mathbf{for}\ \mathbf{0} \le \boldsymbol{\tau} < \boldsymbol{\tau}_{\mathrm{r}} \\ \boldsymbol{C}_{-\mathrm{vdl2}}(\boldsymbol{E}_0)^3\boldsymbol{S}/\boldsymbol{T}_0\boldsymbol{I}_0, & \mathbf{for}\ \boldsymbol{\tau}_{\mathrm{r}} \le \boldsymbol{\tau} \end{cases} \quad \text{Equation S22}$$

$$\boldsymbol{\gamma_3} = \begin{cases} \boldsymbol{C}_{\mathrm{vdl3}}(\boldsymbol{E}_0)^4\boldsymbol{S}/\boldsymbol{T}_0\boldsymbol{I}_0, & \mathbf{for}\ \mathbf{0} \le \boldsymbol{\tau} < \boldsymbol{\tau}_{\mathrm{r}} \\ \boldsymbol{C}_{-\mathrm{vdl3}}(\boldsymbol{E}_0)^4\boldsymbol{S}/\boldsymbol{T}_0\boldsymbol{I}_0. & \mathbf{for}\ \boldsymbol{\tau}_{\mathrm{r}} \le \boldsymbol{\tau} \end{cases} \quad \text{Equation S23}$$

Additionally:

$$\boldsymbol{\epsilon}_{\mathrm{r}} = \boldsymbol{\epsilon} - \boldsymbol{\rho}\boldsymbol{i} \quad \text{Equation S24}$$

$$\boldsymbol{\epsilon} = \boldsymbol{\epsilon}_{\mathbf{dc}} + \boldsymbol{\Delta\epsilon}\mathbf{sin}(\boldsymbol{w\tau} + \boldsymbol{\eta}) \quad \text{Equation S25}$$

$$\boldsymbol{\epsilon}_{\mathbf{dc}} = \begin{cases} \boldsymbol{\epsilon}_{\mathbf{start}} + \boldsymbol{\tau} & \mathbf{for}\ \mathbf{0} \le \boldsymbol{\tau} < \boldsymbol{\tau}_{\mathrm{r}} \\ \boldsymbol{\epsilon}_{\mathbf{reverse}} + \boldsymbol{\tau}_{\mathrm{r}} - \boldsymbol{\tau} & \mathbf{for}\ \boldsymbol{\tau}_{\mathrm{r}} \le \boldsymbol{\tau} \end{cases} \quad \text{Equation S26}$$

Where the dimensionless uncompensated resistance $\rho = R_{\mathrm{u}}I_0/E_0$, $\tau_{\mathrm{r}} = t_{\mathrm{r}}/T_0$, and any ac input signal is parameterized by its dimensionless amplitude $\Delta\epsilon = \Delta E/E_0$, angular frequency $w = \omega T_0$, and phase η. Note the conditions on Equation S20 to Equation S26 depend on the sign of T_0; they are therefore defined above for positive v as this is the standard why of defining such equations, however negative v and therefore equations with the relative equality changes were used in this work.

4.2 Model for one-concerted two-electron transfer

To model Reaction S3, capacitance is as before (Equation S11), and k_3^{red} and k_3^{ox} are calculated from Equation S1 and Equation S2, noting that n = 2. Defining θ_{X} and θ_{Z} as the proportion of the X and Z respectively with initial conditions $\theta_{\mathrm{X}}(0) = 1$ and $\theta_{\mathrm{Z}}(0) = 0$ the ordinary differential equation governing the systems behaviour is given by:

$$\frac{\mathrm{d}\theta_{\mathrm{X}}}{\mathrm{d}t} = k_3^{\mathrm{ox}}(1-\theta_{\mathrm{X}}) - k_3^{\mathrm{red}}\theta_{\mathrm{X}} \quad \text{Equation S27}$$

Note the substitution $\theta_{\mathrm{Z}} = 1 - \theta_{\mathrm{X}}$ has been used and $\frac{\mathrm{d}\theta_{\mathrm{Z}}}{\mathrm{d}t}$ has been omitted as $-\frac{\mathrm{d}\theta_{\mathrm{X}}}{\mathrm{d}t} = \frac{\mathrm{d}\theta_{\mathrm{Z}}}{\mathrm{d}t}$, it is therefore not independent and the system may be fully described without it.

The Faradaic term is due to the electrons transferred between the enzyme and the electrode, and is therefore formulated as:

$$I_{\mathrm{f}} = 2FS\Gamma\left(\frac{\mathrm{d}\theta_{\mathrm{X}}}{\mathrm{d}t}\right) \quad \text{Equation S28}$$

The dimensionless equations using the same parameters as before (see Dimensionless equations) but with the non-dimensionality constants:

$$E_0 = \frac{RT}{2F}, \qquad T_0 = \frac{E_0}{v}, \qquad I_0 = \frac{FS\Gamma_0}{T_0} \quad \text{Equation S29}$$

are therefore:

$$\frac{\mathrm{d}\theta_{\mathrm{X}}}{\mathrm{d}\tau} = \kappa_3^0\left((1-\theta_{\mathrm{X}})e^{(1-\alpha_3)(\epsilon_{\mathrm{r}}-\epsilon_3^0)} - \theta_{\mathrm{X}}e^{-\alpha_3(\epsilon_{\mathrm{r}}-\epsilon_3^0)}\right) \quad \text{Equation S30}$$

and:

$$i = (\gamma + \gamma_1\epsilon_{\mathrm{r}} + \gamma_2\epsilon_{\mathrm{r}}^2 + \gamma_3\epsilon_{\mathrm{r}}^3)\frac{\mathrm{d}\epsilon_{\mathrm{r}}}{\mathrm{d}\tau} + 2\zeta\left(\frac{\mathrm{d}\theta_{\mathrm{X}}}{\mathrm{d}\tau}\right) \quad \text{Equation S31}$$

5 Computational methods

The same two-step fitting approach described in our previous H2ase-MFHypD work was used,[1] first fitting for capacitance in the time domain in regions of the current trace with little or no Faradaic contribution, then fitting the Faradaic parameters in the frequency domain using Fourier transformation. Three models were therefore written, one to simulate and fit only capacitance-current using the equations outlined above and setting I_f to zero, and two full models to simulate both current components and fit for Faradaic parameters in Fourier space using either a model for two-stepwise one-electron transfers ($n_1 = n_2 = 1$) or one-concerted two-electron transfer ($n_3 = 2$). The known experimental parameters are as summarised in Figure S4.

$E_{\text{start}} = -50 - 50(pH - 4)$ mV	$f = 8.977950$ Hz	$\Delta E = 150$ mV	$\alpha_1 = 0.5$
$E_{\text{reverse}} = -650 - 50(pH - 4)$ mV	$T = 298.15$ K	$s = 0.03$ cm^2	$\alpha_2 = 0.5$
$\Gamma_0 = 6.5 \times 10^{-12}$ mol. cm^{-2}	$v = -22.3517$ mV. s^{-1}	$\alpha_3 = 0.5$	

Figure S4 Known parameters of all experimental data, pH in the formulae of E_{start} and E_{reverse} refer to the pH of the experimental data, $\alpha_1$and α_2 are part of the two sequential one electron transfer model ($n_1 = n_2 = 1$), while α_3 is part of the concerted two electron transfer model ($n_3 = 2$).

Prior to fitting, the raw experimental data used for this work was manipulated in two ways: it was down-sampled so that approximately 200 measurements occurred per period of the applied AC current as is standard practice ($\sim$217 was used); and it was ensured that the measurements finished on an integer number of periods as this guarantees the data begins and ends at the same location with respect to the oscillating input signal, easing Fourier transformation. A single period was therefore dropped from the end of all experimental measurements, this had the added benefit of removing larger noisy current spikes present at the end of some of the data. As the same manipulation was done to each experimental current trace fair comparison can be done between experiments. Note that a higher sample rate of $\sim$543 measurements per period was used for plots.

5.1 Forward problem

To solve and code these models the dimensionless equations must be solved for the unknown time dependent variables θ_{X}, θ_{Z}, and i. The same approach was taken as used in our previous work. Firstly, the time derivatives $\frac{\mathrm{d}\theta_{\mathrm{X}}}{\mathrm{d}t}$, $\frac{\mathrm{d}\theta_{\mathrm{Z}}}{\mathrm{d}t}$, and $\frac{\mathrm{d}i}{\mathrm{d}t}$ were discretised using the backwards Euler method. These discretised equations were then substituted into Equation S19/Equation S31, giving an implicit equation in terms of $\theta_{\mathrm{X}}^{\mathrm{n+1}}$, $\theta_{\mathrm{X}}^{\mathrm{n}}$, $\theta_{\mathrm{Z}}^{\mathrm{n+1}}$, $\theta_{\mathrm{Z}}^{\mathrm{n}}$, $i^{\mathrm{n+1}}$, and i^{n}. This was then solved using the Newton-Raphson method which was implemented in Python, thus solving the forward problem. An equivalent approach was taken for the $n_3 = 2$ model. Note that the only discretization required for the capacitance model is $\frac{\mathrm{d}i}{\mathrm{d}t}$, as only Equation S11 or the first term of Equation S19/Equation S31 when non-dimensionalized is needed for the capacitance only model.

5.2 Inverse problem

As previously mentioned, a two-step fitting method, very similar to that used in our previous work on H2ase-MFHypD (Adamson et al., 2017b), was used to fit the experimental data. Firstly, background capacitive current was fitted using a capacitance only model and regions of the experimental data which could be safely described using this model. Subsequently, Faradaic current was fitted for using a Fourier transform to separate the Faradaic signal from the data. This process is outlined below. Note the optimisation in this work was carried out in a Bayesian framework, the mathematics of this has been covered in the electrochemistry literature (Gavaghan et al., 2018; Gundry et al., 2021), with extensive further cover available in the statistical literature (Lambert, 2018), and is therefore not repeated in this work.

5.2.1 Finding best fit parameter values for the non-Faradaic capacitance-current model

Fitting of the background capacitance was carried out in the time domain, using the regions of the experimental data that could be described using only the capacitance model. These regions were identified by visual inspection of the current trace and Figure S5 shows them highlighted in blue for a sample of pH 6.0 experimental data. These regions represent the same time-window across all the data because in the Experimental data collection E_{start} and E_{rev} were adjusted with pH so that the Faradaic region occupied the same time-window at all pH's (see Figure S3). The capacitance parameters C_{vdl}, C_{vdl1}, C_{vdl2}, C_{vdl3}, $C_{-\mathrm{vdl}}$, $C_{-\mathrm{vdl1}}$, $C_{-\mathrm{vdl2}}$, and $C_{-\mathrm{vdl3}}$ were fitted for as well as ω, η, and R_{u}. The phase shift, η found for capacitance was not used for the Faradaic fitting and was refitted for, this is standard practise due to the sensitivity of the model to the phase.

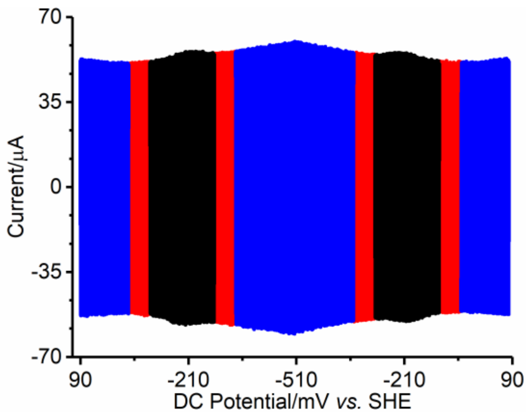

Figure S5 The current trace for pH 6.0 experiment 3, shaded in blue are the assumed capacitive regions used to fit the capacitance parameters and uncompensated resistance, and shaded in black is the voltage region within which E^0_{app1}, E^0_{app2}, and E^0_{app} are fitted for.

This inverse problem was addressed using the infrastructure provide by the PINTS Python library (Clerx et al., 2019). The model was wrapped as a 'Pints.ForwardModel' to allow use of the automated methods of parameter fitting implemented in the PINTS Python library (Clerx et al., 2019). The problem was then optimised using the CMA-ES optimiser, a uniform prior with box bound parameter given in Figure S6 (transformed using 'Pints.RectangularBoundriesTranformation' to aid fitting), and sum of square difference objective function, ℓ_t, between simulated capacitance current, i_{c}, and experimental current, i_{e}, as was done previously (Adamson et al., 2017b).

$$\boldsymbol{\ell_t} = \sum_{\mathbf{j=1}}^{\mathbf{N}} \left[\boldsymbol{i}_{\mathbf{c}}(\boldsymbol{\tau}_{\mathbf{j}}) - \boldsymbol{i}_{\mathbf{e}}(\boldsymbol{\tau}_{\mathbf{j}})\right]^{\mathbf{2}}.$$ Equation S32

For each experimental dataset 10 optimisations were performed using the box bound parameters described in Figure S6, the objective function given in Equation S32, and the CMA-ES optimiser from the PINTS Python library (Clerx et al., 2019). A maximum number of iterations of 10,000, or 200 consecutive unchanged iterations with a threshold of 1e-11 being deemed as unchanged, was used to terminate each optimisation. The fit giving the lowest objective functions score was deemed the best and was generated a minimum of 3 times in each experiment. The results of this are presented in Table S4.

0≤ C_{dl} ≤1e-3 , (F cm^{-2})	-1e-3≤ C_{dl1} ≤ 1e-3 , (F cm^{-2} V^{-1})	-1e-3≤ C_{dl2} ≤1e-3 , (F cm^{-2} V^{-2})	-1e-3≤ C_{dl3} ≤1e-3 , (F cm^{-2} V^{-3})
0≤ $C_{-\mathrm{dl}}$ ≤1e-3 , (F cm^{-2})	-1e-3≤ $C_{-\mathrm{dl1}}$ ≤ 1e-3 , (F cm^{-2} V^{-1})	-1e-3≤ $C_{-\mathrm{dl2}}$ ≤1e-3 (F cm^{-2} V^{-2}),	-1e-3≤ $C_{-\mathrm{dl3}}$ ≤1e-3 , (F cm^{-2} V^{-1})
$0.99\omega_0 \leq \omega \leq 1.01\omega_0$, (rad s^{-1})	-0.15$\pi \leq \eta \leq$ 0.15π , (rad)	0.1 ≤ R_{u} ≤ 140 , (Ω)	

Figure S6 Box bound prior distribution of capacitance parameters for inference, with $\omega_0 = 2\pi f$.

5.2.2 Finding best fit parameter values for the two different reaction models

Fourier transformation is a very natural way of transforming the output of an AC voltammetry experiment, as the constant frequency AC nature of the input signal gives a natural timescale to the output. Indeed it has been shown in the literature that fourth and higher order harmonics form PF-FTACV have negligible capacitive current and therefore provide access to an essentially background free measurement of Faradaic current derived from fast protein redox processes (Lee et al., 2011; Stevenson et al., 2012; Adamson et al., 2017a). As in our previous work on H2ase-MFHypD data we took advantage of this separation using a two-step fitting process (Adamson et al., 2017b), firstly fitting capacitance parameters as outlined above, before determining the Faradaic parameters that define protein redox processes ($k^0_{app\ \mathrm{i}}$, $E^0_{app\ \mathrm{i}}$, η , and ζ) by fitting to the real and imaginary contributions of the 4th to 12th order harmonics of the ac data using data optimisation methods. Harmonics 4 to 12 are used as harmonics below the 4th describe capacitance and harmonics above the 12th (for our data) become masked by experimental noise.

Fourier transformation of the experimental and simulated data was therefore carried out using the fast Fourier transform available in the NumPy Python library (Harris et al., 2020). Additional manipulations of the experimental data and simulated data was then carried out to separate out the 4th to 12th harmonics. The Fourier transformed data corresponding to $3.5f$ to $12.5f$ was used to 'top hat' filter for the harmonics, this generous range either side of the peripheral harmonics was used to account for any phase shift and/or inaccuracy in the experimental reported frequency.

The infrastructure provide by the PINTS Python library was again employed for optimisation, and the model was therefore wrapped as a 'Pints.ForwardModel' (Clerx et al., 2019). The

problem was then optimised using the C-MAES optimiser, a uniform prior with box bound parameter given in Figure S7 (transformed using 'Pints.RectangularBoundriesTranformation' to aid fitting), and an Euclidean distance objective function, ℓ_{fq}, between simulated Fourier transformed current, $f_{\mathrm{n}}(w_{\mathrm{k}})$, and Fourier transformed experimental current, $f_{\mathrm{e}}(w_{\mathrm{k}})$:

$$\boldsymbol{\ell}_{fq} = \sqrt{\frac{\sum_{\mathbf{k}}^{\mathbf{N}} |\boldsymbol{f}_{\mathbf{n}}(\boldsymbol{w}_{\mathbf{k}}) - \boldsymbol{f}_{\mathbf{e}}(\boldsymbol{w}_{\mathbf{k}})|^2}{\mathbf{N}}} \quad \text{Equation S33}$$

Here w_{k} is frequency, and note the use of the modulus operator as $f_{\mathrm{n}}(w_{\mathrm{k}})$ and $f_{\mathrm{e}}(w_{\mathrm{k}})$ are complex numbers, this transforms the resultant complex number into a real number describing the magnitude of the complex number allowing minimisation of the difference. This differs to the objective function used in our previous work in three ways: in this work a 'top hat' filter of range $3.5f$ to $12.5f$ was used to separate harmonics 4-12 and the entire range was used in the objective function as opposed to applying a Kaiser window for each individual harmonic; the objective function is normalised by N; and no windowing functions were used.

$0.0 \leq k^0_{app\,1} \leq 4000$, (s^{-1})	$0.0 \leq k^0_{app\,2} \leq 4000$ (s^{-1}),
$E_{\min} \leq E^0_{app\,1} \leq E_{\max}$, (mV)	$E_{\min} \leq E^0_{app\,2} \leq E_{\max}$, (mV)
$-0.314 \leq \eta \leq 0.314$, (rad)	$0.0 \leq \zeta \leq 10.0.$

$0.0 \leq k^0_{app\,3} \leq 4000$, (s^{-1})	$E_{\min} \leq E^0_{app\,3} \leq E_{\max}$, (mV)
$-0.314 \leq \eta \leq 0.314$, (rad)	$0.0 \leq \zeta \leq 10.0.$

Figure S7 Box bound uniform distributions of Faradaic parameters for inference, where $E_{\min} = E_{\mathrm{starts}} + 0.375(E_{\mathrm{reverse}} - E_{\mathrm{start}})$, $E_{\min} = E_{\mathrm{starts}} + 0.575(E_{\mathrm{reverse}} - E_{\mathrm{start}})$, and $\zeta = \Gamma/\Gamma_0$ as before. On the left are the 6 parameters fitted for when using the two-sequential one-electron transfer model ($n_1 = n_2 = 1$) and on the right are the 4 parameters fitted for when using the concerted two-electron transfer model ($n_3 = 2$). An example of the fitting range described by $E_{\min}$ and $E_{\max}$ can be seen in Figure S5 (black trace).

10 optimisations were performed using the box bound parameters described in Figure S7, the objective function given in Equation S33, and the CMA-ES optimiser from the PINTS Python library for each experimental current trace (Clerx et al., 2019). A maximum number of iterations of 10,000 or 200 consecutive unchanged iterations with a threshold of 1e-11 being deemed as unchanged was used to terminate each optimisation. The fit giving the lowest objective functions score was deemed the best and was generated a minimum of 4 times in each experiment. The results of this are discussed in the main paper, with results of fitting to the n_1=n_2=1 model given in Table 1, and results of fitting to the n=2 model given in Table S3. The best fit capacitance parameters previously determined in the first fitting step were used for each data set. Note the phase parameter, η, from capacitance fitting was not used and was fitted for again due to a shift that occurs between capacitive and Faradaic

regions, as was done in our previous work (Adamson et al., 2017b). The box bound parameters described in Figure S7 were chosen to be consistent with our previous work on H2ase-MFHypD (Adamson et al., 2017b). However, a narrower fitting range for E^0_{app1}, E^0_{app2}, and E^0_{app3} was used as this can be seen to be physically sensible in Figure S5 (black shaded region).

5.3 Verification of computational methods

All models and inference methods used in this study were verified before use on experimental data. This section details the verification process.

5.3.1 Forward problem verification

The solutions to the $n_3 = 2$ and $n_1 = n_2 = 1$ models detailed in Forward Problem were verified by taking an additional approach involving recasting Equation S19/Equation S31 as an ordinary differential equation (ODE) and solving the resulting system of ODE's using the ODE solver 'odeint' from the SciPy Python library using default tolerances - positive error weights of 1.49E-8 (Virtanen et al., 2020). The two implementations of the models agreed - weighted mean square differences of order 1.0E-16 A^2 - across all parameter sets tested verifying that the Newton-Raphson model was correctly implemented. Note the Newton-Raphson approach is preferred for electrochemical problems as it is substantially faster to solve. Additionally, the forward model was verified by reproducing result figures from the literature (Stevenson et al., 2012).

5.3.2 Inverse problem verification

The implementation of the objective function and inference methods for the capacitance and faradaic models were verified by fitting to synthetic data using the experimental relevant parameters given in Figure S4, capacitance and faradaic parameters within the ranges given in Figure S6 and Figure S7 respectively. Both capacitance and faradaic models could reproduce all parameters within the range shown in Figure S6 and Figure S7 respectively without experimental noise. Faradaic parameters were regenerated exactly (to a minimum of 11 S.F) giving an objective function score of ~0.0 (1E-8). Capacitance parameters were fitted for to synthetic data generated using the capacitance model allowing exact recover (to a minimum of 13 S.F) giving an objective function score of ~0.0 (1E-19). Additionally, capacitance parameters were fitted for to synthetic data generated using the full faradaic model to better emulate fitting to experimental data; an objective function of zero was therefore not achieved but the capacitance parameters were regenerated to 2-11 S.F; the fitted capacitance parameters were then used to fit the faradaic parameters yielding an objective function score of 47.8 and parameters accurate to 3-5 S.F. Thus verifying the implementations were working correctly, and preforming as expected.

6 Comparing best fit objective function values for the two models

A lower objective function score (Equation S33) indicates a better fit between a simulated dataset and experimental data. As the two different reaction models ($n_1=n_2=1$ versus $n_3=2$) use different non-dimensionalization constants, the objective function scores generated from the "best fit" parameter sets are converted into their dimensional form and then compared. Table S1 shows scores for pH 4.0, pH 5.0, and pH 6.0 data while Table S2 shows the equivalent values for pH 7.0, pH 8.0, and pH 9.0. This analysis emphasises that the $n_1=n_2=1$ model provides a better fit to the data for all experiments at all pH's studied.

Table S1 Dimensional objective function values (Equation S33) for the best fit parameter values for the simultaneous two-electron transfer model ($n_3=2$, see Table S3) and the two-sequential one-electron transfer model ($n_1=n_2=1$, Table 1) for three v = 22.4 mV s^{-1}, f = 8.96 Hz, ΔE = 150 mV H2ase-MFHypD FTACV experiments at pH 4.0, 5.0, and 6.0. The objective function has been reported in dimensional units to allow comparison between the two models. The better fit is shaded in for each experiment.

pH		4.0			5.0			6.0		
Experiment		1	2	3	1	2	3	1	2	3
$\ell_{fq}(x)$ (10^{-4} As)	n=2	2.84	2.76	2.77	2.83	2.73	2.77	4.07	2.86	2.85
	$n_1=n_2=1$	2.71	2.65	2.68	2.73	2.61	2.63	3.98	2.76	2.75

Table S2 Dimensional objective function values (Equation S33) for the best fit parameter values for the simultaneous two-electron transfer model ($n_3=2$, see Table S3) and the two-sequential one-electron transfer model ($n_1=n_2=1$, Table 1) for three v = 22.4 mV s^{-1}, f = 8.96 Hz, ΔE = 150 mV H2ase-MFHypD FTACV experiments at pH 7.0, 8.0, and 9.0. The objective function has been reported in dimensional units to allow comparison between the two models. The better fit is shaded in for each experiment.

pH		7.0			8.0			9.0		
Experiment		1	2	3	1	2	3	1	2	3
$\ell_{fq}(x)$ (10^{-4} As)	n=2	3.04	2.87	2.86	3.03	2.76	2.77	3.04	2.84	2.83
	$n_1=n_2=1$	2.95	2.77	2.74	2.95	2.67	2.69	2.92	2.71	2.70

7 Comparing pH 5.0, 6.0, 7.0 and 8.0 experimental data and best fit plots from both reaction models

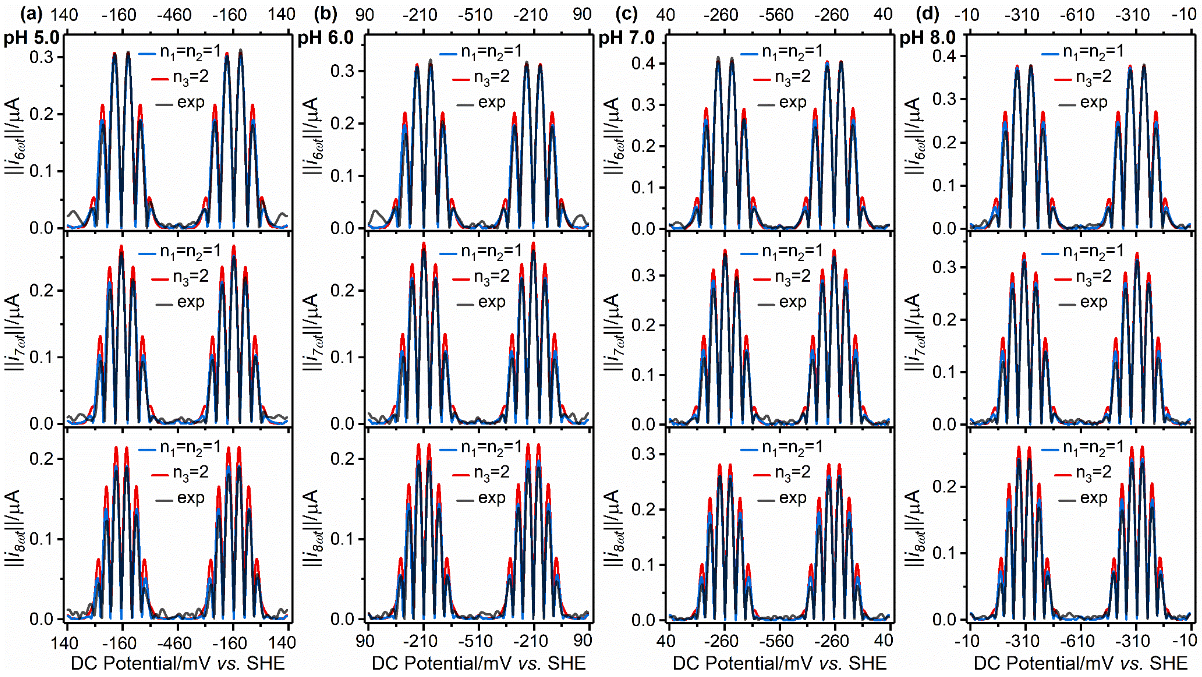

Figure S8 Overlay current-potential plots of (a) pH 5.0, (b) pH 6.0, (c) pH 7.0, and (d) pH 8.0 (grey line) data from experiment 3, and best fit simulated data using (red line) a concerted two-electron (n_3 = 2) electron transfer model and (blue line) a stepwise two-sequential one-electron transfer reaction model (n_1=n_2=1) for (top) 6th, (middle) 7th, and (bottom) 8th harmonics. The parameter values used to generate the simulations are reported in Table 1, Table S3 and Table S4.

8 Best fit parameter values for the one-concerted two-electron transfer model

Table S3 Best fit parameter values extracted when the simultaneous two electron transfer model is used to simulate three v = 22.4 mV s^{-1}, f = 8.96 Hz, ΔE = 150 mV H2ase-MFHypD FTACV experiments at each of the pH values 4.0, 5.0, 6.0, 7.0, 8.0 and 9.0. The rate constant, k^0_{app1}, and the objective function (Equation S33) are reported to 4 S.F, all other parameters are reported to 3 S.F.

pH	Exp	k^0_{app3} (s^{-1})	E^0_{app3} (mV *vs.* SHE)	η (rad)	Γ ($p$mol cm^{-2})	$\ell_{fq}(x)$ (10^3)
4.0	1	133.1	-95.6	1.45e-1	2.49	8.687
	2	129.0	-95.4	1.36e-1	2.50	8.448
	3	132.3	-95.6	1.34e-1	2.49	8.474
5.0	1	129.6	-153	1.65e-1	2.67	8.643
	2	128.4	-152	1.42e-1	2.67	8.338
	3	138.3	-152	1.32e-1	2.60	8.453
6.0	1	145.9	-210	1.68e-1	2.67	12.42
	2	129.3	-210	1.64e-1	2.69	8.741
	3	142.4	-210	1.47e-1	2.65	8.699
7.0	1	225.5	-262	1.52e-1	3.42	9.298
	2	182.3	-261	1.69e-1	3.49	8.760
	3	196.3	-261	1.58e-1	3.42	8.738
8.0	1	280.1	-301	1.50e-1	3.17	9.244
	2	208.4	-301	1.70e-1	3.24	8.428
	3	196.5	-301	1.71e-1	3.22	8.473
9.0	1	217.5	-337	1.41e-1	3.39	9.296
	2	203.2	-337	1.35e-1	3.39	8.668
	3	204.4	-337	1.28e-1	3.37	8.633

9 Best fit capacitance parameter values

As detailed above, in finding best fit parameter values for the non-Faradaic capacitance-current model the parameters C_{vdl}, C_{vdl1}, C_{vdl2}, C_{vdl3}, $C_{-\mathrm{vdl}}$, $C_{-\mathrm{vdl1}}$, $C_{-\mathrm{vdl2}}$, $C_{-\mathrm{vdl3}}$ were fitted for as well as ω, η, and R_{u}. The resulting bast fit parameter values for all experimental data are summarised in Table S4. To illustrate the accuracy of the non-Faradaic capacitance-current model in fitting to the background current of the experimental data, Figure S9 shows an overlay of a pH 6.0 experimental dataset and the corresponding simulation of the capacitance using the best-fit parameter values.

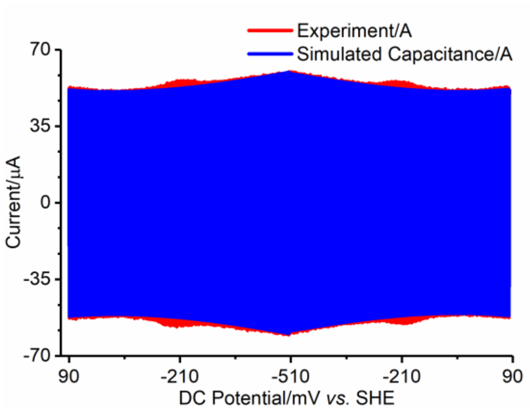

Figure S9 (Red) The current trace for pH 6.0 experiment 3, and (blue) the corresponding simulation of the capacitance using the best-fit parameter values reported in Table S4.

Table S4 Best fit capacitance parameter values found when simulating three repeated $v = 22.4$ mV s^{-1}, $f = 8.96$ Hz, $\Delta E = 150$ mV H2ase-MFHypD FTACV experiments at each of the pH values 4.0, 5.0, 6.0, 7.0, 8.0 and 9.0 using the approach detailed in the Computational methods section and the capacitance model detailed in Mathematical model section of the SI. All parameters are reported to 3 S.F.

pH	Exp	C_{vdl}<br>$C_{-\mathrm{vdl}}$<br>(F cm^{-2})	C_{vdl1}<br>$C_{-\mathrm{vdl1}}$<br>(F cm^{-2} V^{-1})	C_{vdl2}<br>$C_{-\mathrm{vdl2}}$<br>(F cm^{-2} V^{-2})	C_{vdl3}<br>$C_{-\mathrm{vdl3}}$<br>(F cm^{-2} V^{-3})	ω<br>(rad s^{-1})	f (Hz)	η (rad)	R_{u} (Ω)
4.0	1	1.86e-04<br>1.89e-04	1.38e-04<br>1.69e-04	3.76e-04<br>4.33e-04	2.06e-04<br>2.41e-04	5.63e+01	8.96	-1.014e-01	33.0
	2	1.88e-04<br>1.89e-04	1.62e-04<br>1.74e-04	4.18e-04<br>4.41e-04	2.27e-04<br>2.45e-04	5.63e+01	8.96	-1.09e-01	14.7
	3	1.88e-04<br>1.90e-04	1.64e-04<br>1.74e-04	4.22e-04<br>4.40e-04	2.30e-04<br>2.44e-04	5.63e+01	8.96	-1.11e-01	9.07
5.0	1	1.95e-04<br>2.03e-04	1.11e-04<br>1.78e-04	3.05e-04<br>4.27e-04	1.45e-04<br>2.21e-04	5.63e+01	8.96	-9.72e-02	49.8
	2	2.01e-04<br>2.03e-04	1.66e-04<br>1.82e-04	4.12e-04<br>4.33e-04	2.14e-04<br>2.26e-04	5.63e+01	8.96	-1.08e-01	18.4
	3	2.01e-04<br>2.03e-04	1.71e-04<br>1.84e-04	4.22e-04<br>4.37e-04	2.21e-04<br>2.291e-04	5.63e+01	8.96	-1.09e-01	14.3
6.0	1	2.05e-04<br>2.20e-04	2.31e-05<br>1.36e-04	6.73e-05<br>2.84e-04	-3.53e-05<br>9.97e-05	5.63e+01	8.96	-9.14e-02	70.9
	2	2.17e-04<br>2.20e-04	1.19e-04<br>1.43e-04	2.53e-04<br>2.94e-04	7.92e-05<br>1.07e-04	5.63e+01	8.96	-9.95e-02	49.1
	3	2.17e-04<br>2.20e-04	1.26e-04<br>1.49e-04	2.70e-04<br>3.06e-04	9.21e-05<br>1.16e-04	5.63e+01	8.96	-1.02e-01	42.0
7.0	1	2.30e-04<br>2.46e-04	-3.36e-05<br>6.72e-05	3.94e-05<br>2.00e-04	-2.89e-05<br>5.64e-05	5.63e+01	8.96	-7.38e-02	95.3
	2	2.43e-04<br>2.46e-04	5.79e-05<br>7.13e-05	1.99e-04<br>2.03e-04	5.98e-05<br>5.89e-05	5.63e+01	8.95932	-7.36e-02	92.9
	3	2.43e-04<br>2.46e-04	6.21e-05<br>7.42e-05	2.04e-04<br>2.07e-04	6.29e-05<br>6.16e-05	5.63e+01	8.96	-7.49e-02	88.9
8.0	1	2.11e-04<br>2.35e-04	-1.63e-04<br>-3.30e-05	-2.09e-04<br>-1.80e-05	-1.67e-04<br>-7.33e-05	5.63e+01	8.96	-6.90e-02	117.4
	2	2.31e-04<br>2.36e-04	-4.56e-05<br>-2.49e-05	-2.48e-05<br>-8.69e-06	-7.33e-05<br>-6.72e-05	5.63e+01	8.96	-7.01e-02	111
	3	2.32e-04<br>2.36e-04	-3.56e-05<br>-2.31e-05	-9.70e-06<br>-8.24e-06	-6.46e-05<br>-6.72e-05	5.63e+01	8.96	-7.24e-02	105
9.0	1	1.69e-04<br>2.11e-04	-3.19e-04<br>-1.10e-04	-4.89e-04<br>-1.97e-04	-3.02e-04<br>-1.67e-04	5.63e+01	8.96	-9.21e-02	79.9
	2	2.03e-04<br>2.13e-04	-1.37e-04<br>-9.70e-05	-2.24e-04<br>-1.80e-04	-1.77e-04<br>-1.57e-04	5.63e+01	8.96	-9.66e-02	62.4
	3	2.06e-04<br>2.14e-04	-1.19e-04<br>-8.86e-05	-1.99e-04<br>-1.68e-04	-1.63e-04<br>-1.51e-04	5.63e+01	8.96	-9.88e-02	55.8

10 Misspecification of the $n_3 = 2$ model

Figure 4 demonstrates that clear differences in FTACV harmonics are observed in the modelling of very fast electrode kinetics, which mimic the responses predicted in the reversible Nernstian regime, and slower regimes, associated with quasi-reversible processes. For reversible electron transfer, the higher order harmonic signals are characterized by well separated lobes, with the current dropping to zero between each lobe. In the slower quasi-reversible kinetic regime, a loss of resolution is observed, which is particularly clear at the outermost lobe (Figure 4).

As shown in Table S3, the best fit parameters obtained for the concerted two-electron transfer (n_3=2) model fall into the slow kinetic regime. Conversely, as visually represented in Figure S10, the experimental data falls into the fast kinetic regime, with the best fits of the n_3=2 model failing to accurately describe the current traces of harmonics 6, 7, and 8. This is supportive of the conclusion that the n_3=2 model is mis-specifed for the H2ase-MFHypD data at all pH's studied.

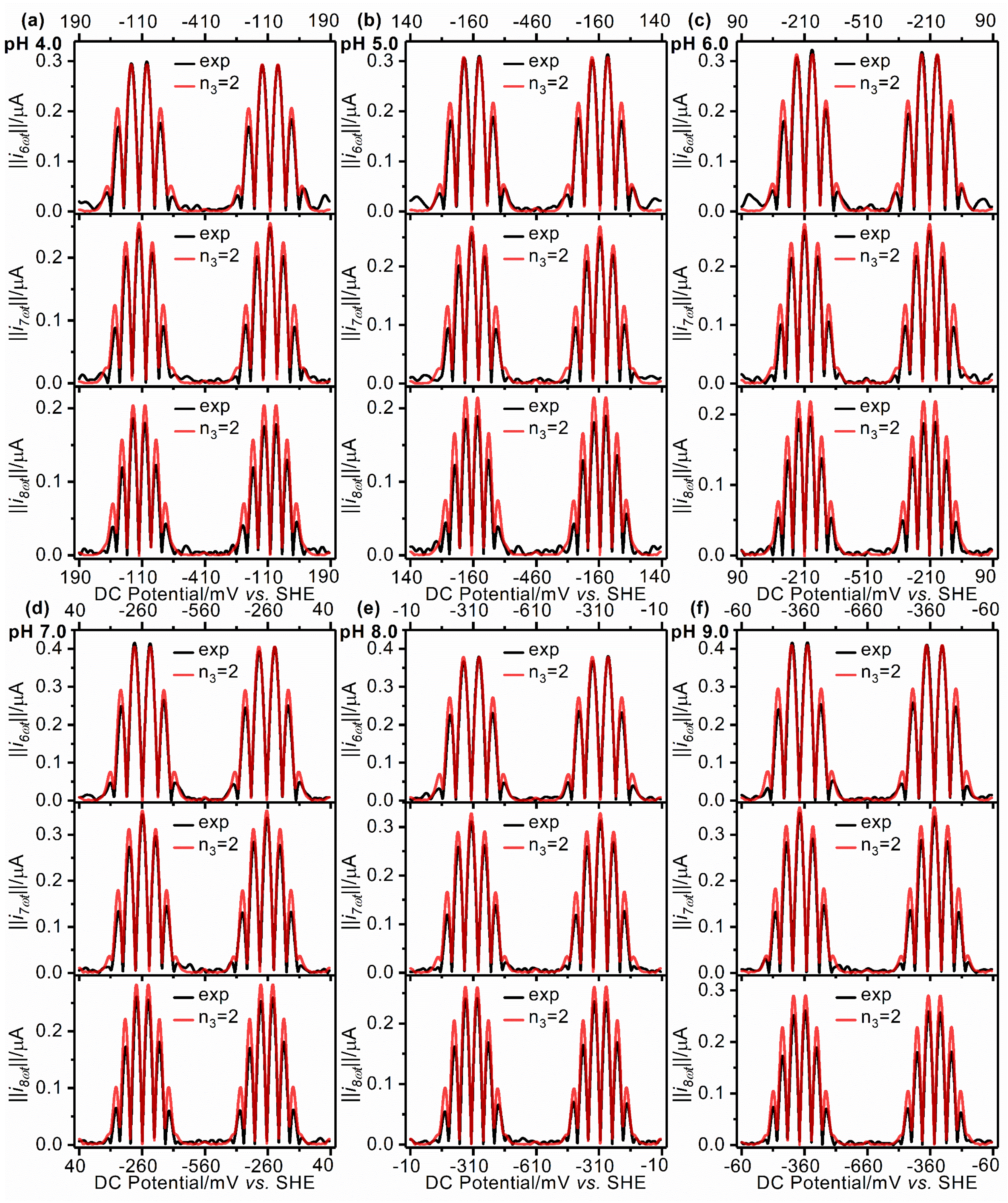

Figure S10 The 6th (top), 7th (middle), and 8th (bottom) harmonics for experiment 3 at (a) pH 4.0, (b) pH 5.0, (c) pH 5.0, (d) pH 6.0, (e) pH 8.0, and (f) pH 9.0 along with harmonics generated from the best fit parameters of the concerted two-electron transfer model to the experimental data reported in Table S3.

11 Verification of H2ase-MFHypD Signal

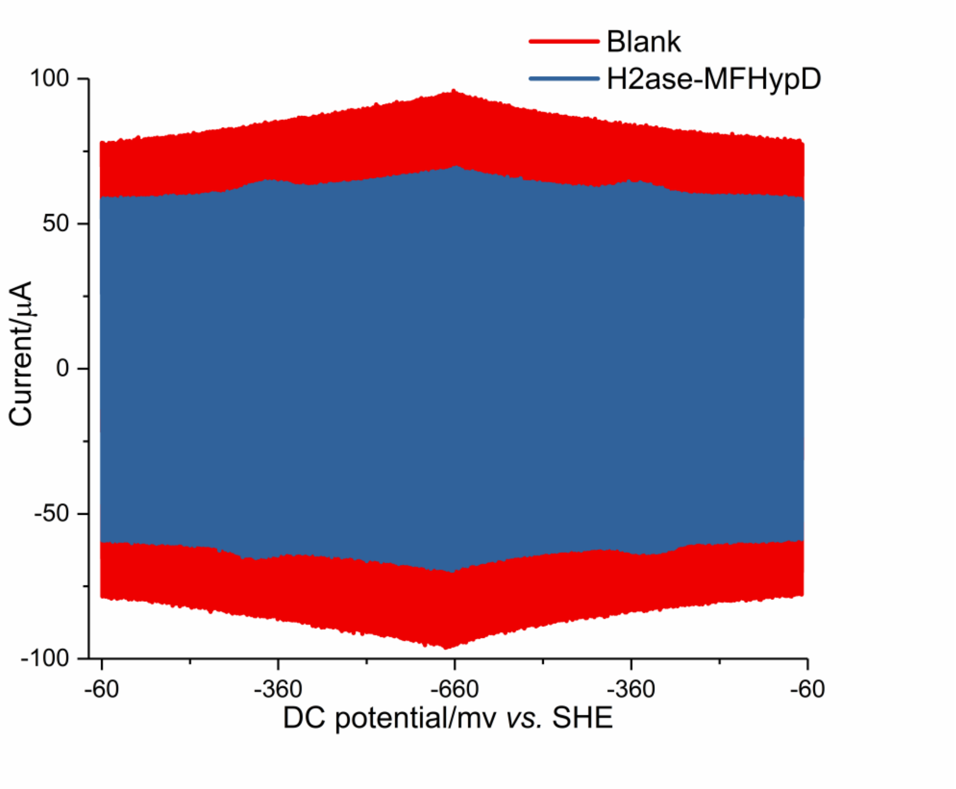

Figure S11 'Blank' electrode (Red) total (AC plus DC) current voltammogram at pH 9.0 for experiment 3, and (blue) the corresponding experimental voltammogram with adhered H2ase-MFHypD.

It is common practice to verify that the faradaic current arises from protein film formation, and is not an artefact, by undertaking voltametric experiments with 'blank' electrodes before and after adhering the protein. The total (AC plus DC) current voltammograms derived from experiments conducted at pH 9.0 before and after protein film formation is shown in Figure S11; this verifies the protein had adhered and the observed faradaic signal results from the protein.

12 Inference and MCMC for E_{app} versus pH

To probe the relationship between the separation of E^0_{app1} and E^0_{app2} with pH we have used the infrastructure of the 'PINTS' Python library to fit the pH vs E_{app} values inferred in this work (Table 1) to Equation 2 and Equation 3 (reproduced here as Equation S34 and Equation S35 recpectively), taken from the literature (Hirst, 2006). The pKa/Ka values of these equations are defined in Figure S12, as are the true reversible potentials, E^0_1 and E^0_2. When fitting the standard deviation of the noise on the reversible potentials $\sigma_{E^0_1}$ and $\sigma_{E^0_2}$ were also inferred, as a Gaussian log likelihood objective function was used.

$$E^0_{app1} = \mathrm{E}^0_1 - \frac{RT}{F}\ln\left[\left(1+\frac{[H^+]}{K_{aB}}+\frac{[H^+]^2}{K_{aA}K_{aB}}\right) \div \left(1+\frac{[H^+]}{K_{aD}}+\frac{[H^+]^2}{K_{aC}K_{aD}}\right)\right] \quad \text{Equation S34}$$

$$E^0_{app2} = \mathrm{E}^0_2 - \frac{RT}{F}\ln\left[\left(1+\frac{[H^+]}{K_{aD}}+\frac{[H^+]^2}{K_{aC}K_{aD}}\right) \div \left(1+\frac{[H^+]}{K_{aF}}+\frac{[H^+]^2}{K_{aE}K_{aF}}\right)\right] \quad \text{Equation S35}$$

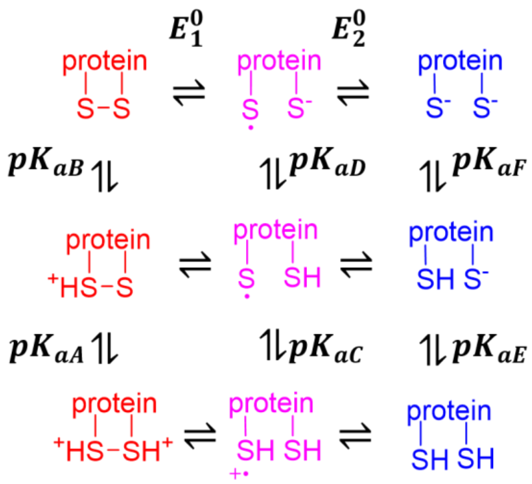

Figure S12 Square scheme from Figure 1(b) updated to define the appropriate pKa and E^0 symbols associated with each protonation and redox reaction, respectively, used to define the voltammetry of H2ase-MFHypD. This is a reproduction of Figure6(a) without arrows to indicated suggested routes across the square scheme.

As with the other inverse problems in this work, the infrastructure provide by the PINTS Python library was used and the model was written in a 'Pints.ForwardModel' class .(Clerx et al., 2019) Twenty optimizations were then performed using the C-MAES optimiser; and a uniform prior transformed by ‘Pints.RectangularBoundriesTranformation’ to aid fitting consistening of the following bounds: all pKa’s were bound between 0.0 and 14, the true reversible potentials, E_1^0 and E_2^0, were bound between -0.3 V and -0.8 V, and the standard deviation of the noise on the reversible potentials, $\sigma_{E_1^0}$ and $\sigma_{E_2^0}$, were bound between 10^{-2} and10^{-8}. For an objective function the following Gaussian log likelihood function, as implemented in the ‘PINTS’ Python library was used:

$$\log L(\boldsymbol{\theta},\boldsymbol{\sigma}|\boldsymbol{x}) = -\frac{n_t n_0}{2}\log 2\pi - \sum_{i=1}^{n_0} n_t \log\sigma_i - \sum_{i=1}^{n_0}\left[\frac{1}{2\sigma_i^2}\sum_{j=1}^{n_0}\left(x_j - f_j(\boldsymbol{\theta})\right)\right] \qquad \text{Equation S36}$$

where $\boldsymbol{\theta}$ is a vector containing the model input parameters, $\boldsymbol{\sigma}$ is a vector of the standard deviation of the noise on the model output parameters (σ_i), $\boldsymbol{x}$ is a vector of the experimental data (x_i) at each pH point, n_t is the number of pH/data points, n_0 is the number of output of the system of equations being analyzed (2 in our case), and $f_i(\boldsymbol{\theta})$ is the simulated data at pH i and input parameters $\boldsymbol{\theta}$**.** The best fit from this fitting process is given in Figure 6(b) and gave an objective function score of 149 to 3 S.F.

Posterior distributions were subsequently obtained using an adaptive Metropolis MCMC algorithm, given as algorithm 4 in (Andrieu and Thoms, 2008) and described in (Haario et al., 2001), as implemented in the ‘PINTS’ Python library as ‘pints.HaarioACMC’ (Clerx et al., 2019). Three chains were used starting at 1.15, 1.1, and 0.9 times the best fit positions previously obtained (Figure 6(b)), chain lengths of 45000, and burn in of 20000 was determined from observation of the chain plots in Figure S13, the autocorrelation plots in Figure S14(b), and the RHAT values reported in Table S5. A uniform prior was used for all parameters covering the same ranges used for optimization and the same log-likelihood function was also used, Equation S36. The obtained posterior distributions are given in Figure S14(a). Additionally, the range of these distributions are shown in Figure 7, where they are wide enough to be shown. Pairwise distributions are shown in Figure S15.

Table S5 RHAT values calculated from the sampling performed using Equations S34 and S35 with a burn in of 20000 samples.

$E_1^0 = 1.0070$	$\sigma_{E_1^0} = 1.0087$	$pK_{aA} = 1.0070$	$pK_{aC} = 1.0132$	$pK_{aE} = 1.0090$
$E_2^0 = 1.0070$	$\sigma_{E_2^0} = 1.0027$	$pK_{aB} = 1.013$	$pK_{aD} = 1.0078$	$pK_{aF} = 1.0069$

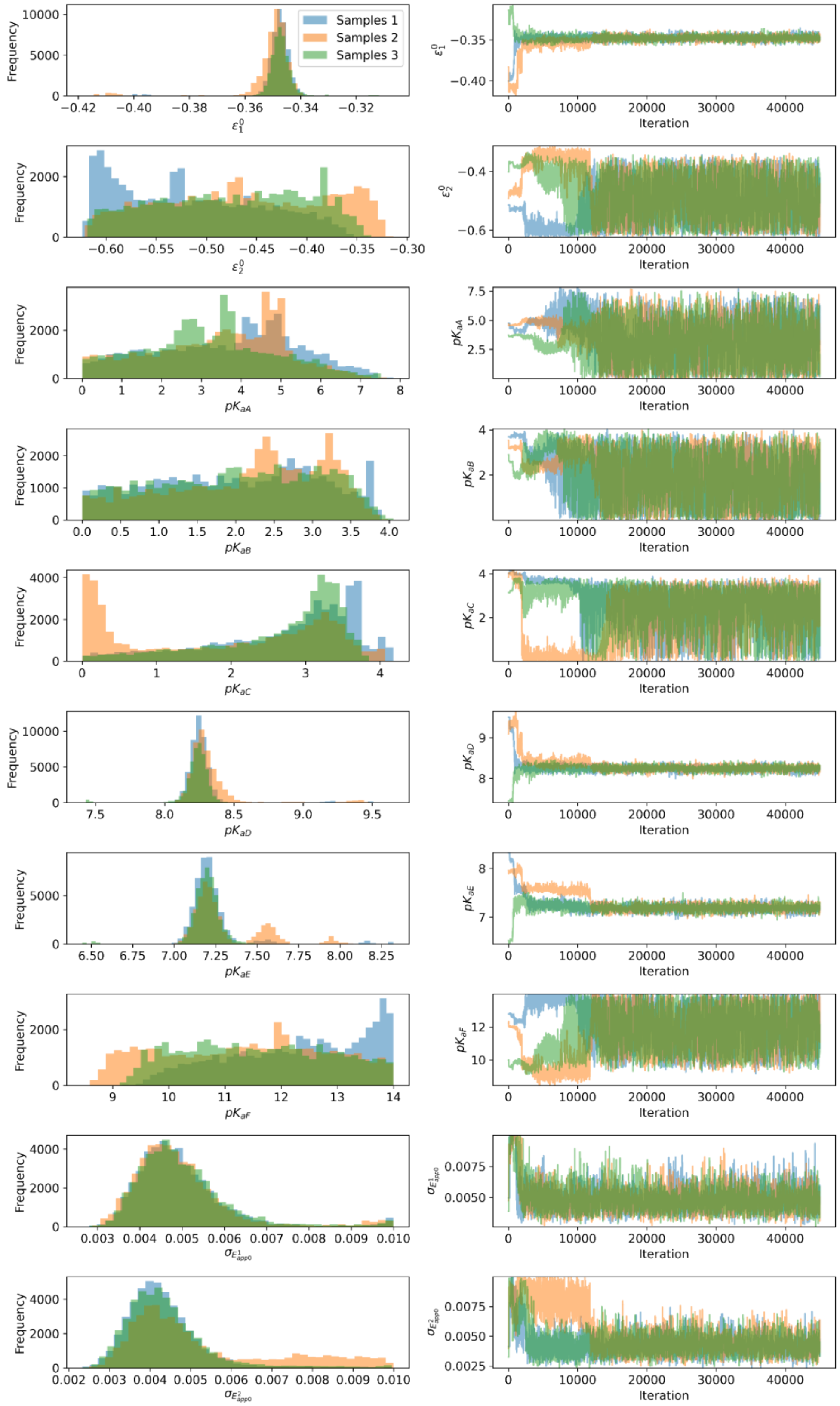

Figure S13 Posterior distributions (left) and MCMC chains (right) form the sampling performed with Equation S34 and Equation S35.

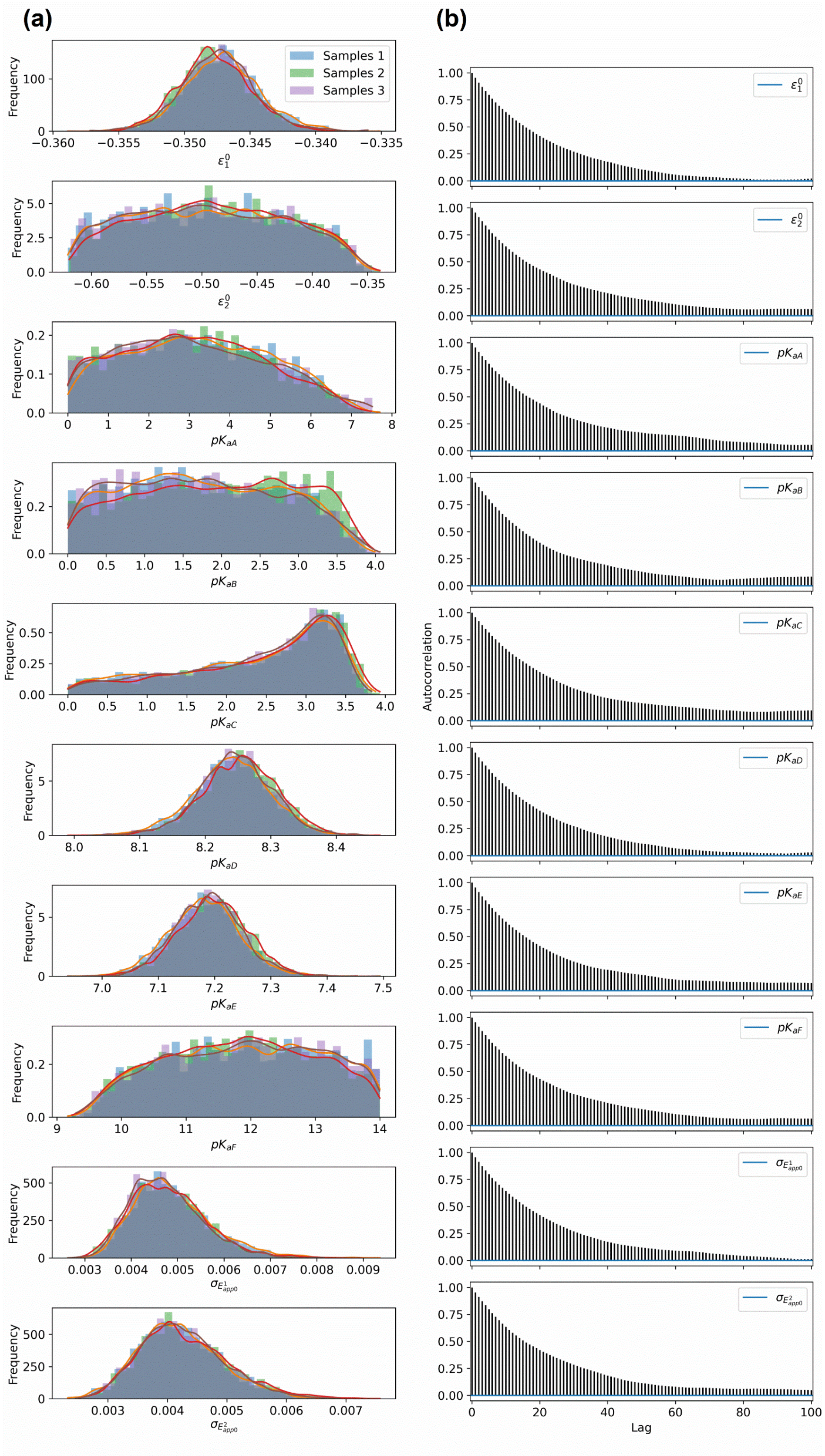

Figure S14 (a) Posterior distribution histograms form the sampling performed with Equation S34 and Equation S35 with burn in discarded (first 20,000; samples from each 45,000 sample chain). (b) Autocorrelation of chain/sample 1, with burn in discarded, 100 lags are plotted.

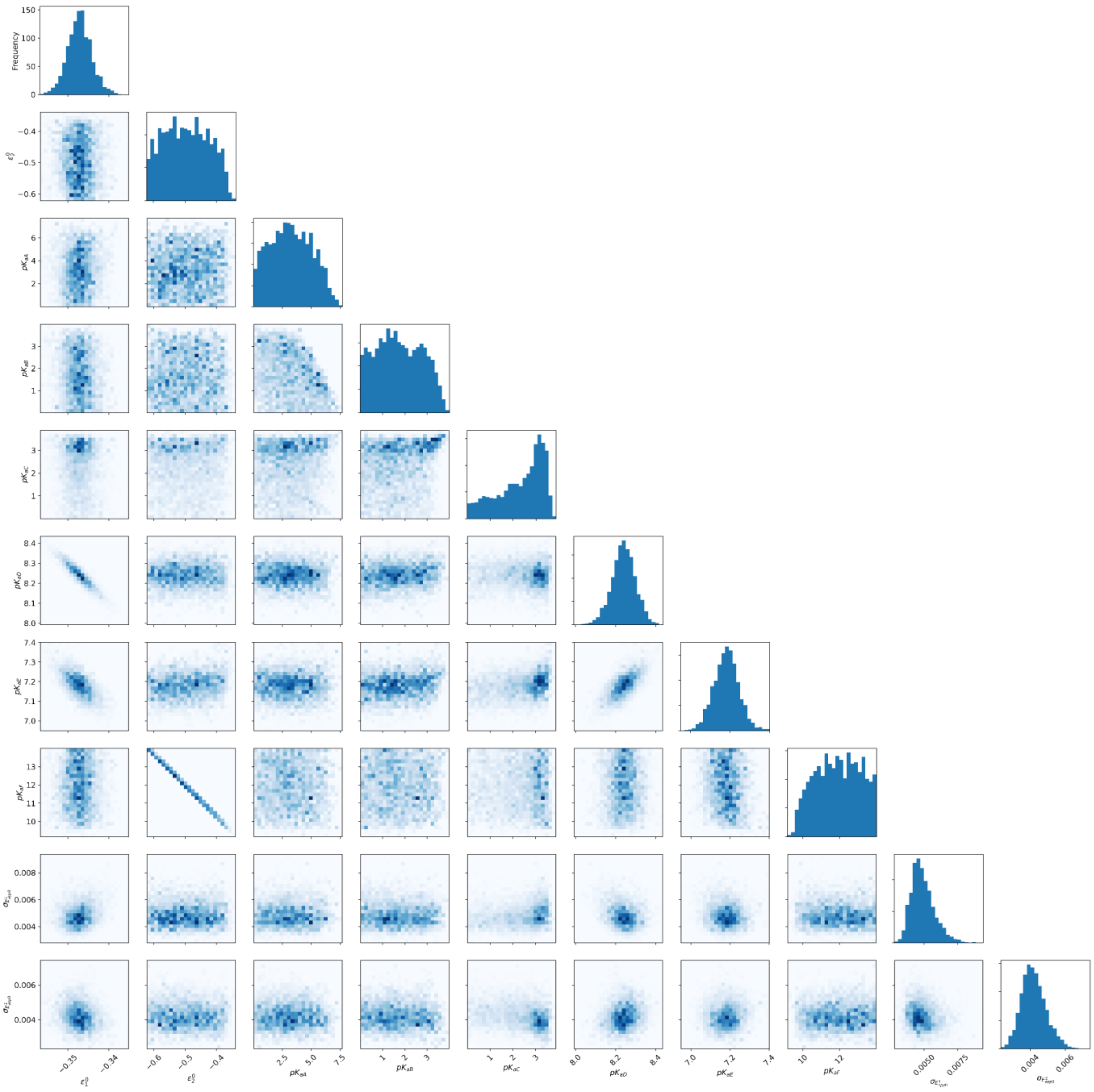

Figure S15 Pairwise plots of the posterior distribution of chain/sample 1 form the sampling performed using Equation S34 and Equation S35 with burn in discarded (first 20,000; samples from each 45,000 sample chain).

13 Insensitivity of Faradaic fitting to Uncompensated Resistance

Figure S16 demonstrates the insensitivity of the faradaic current to uncompensated resistance in designated harmonics and hence our fitting method.

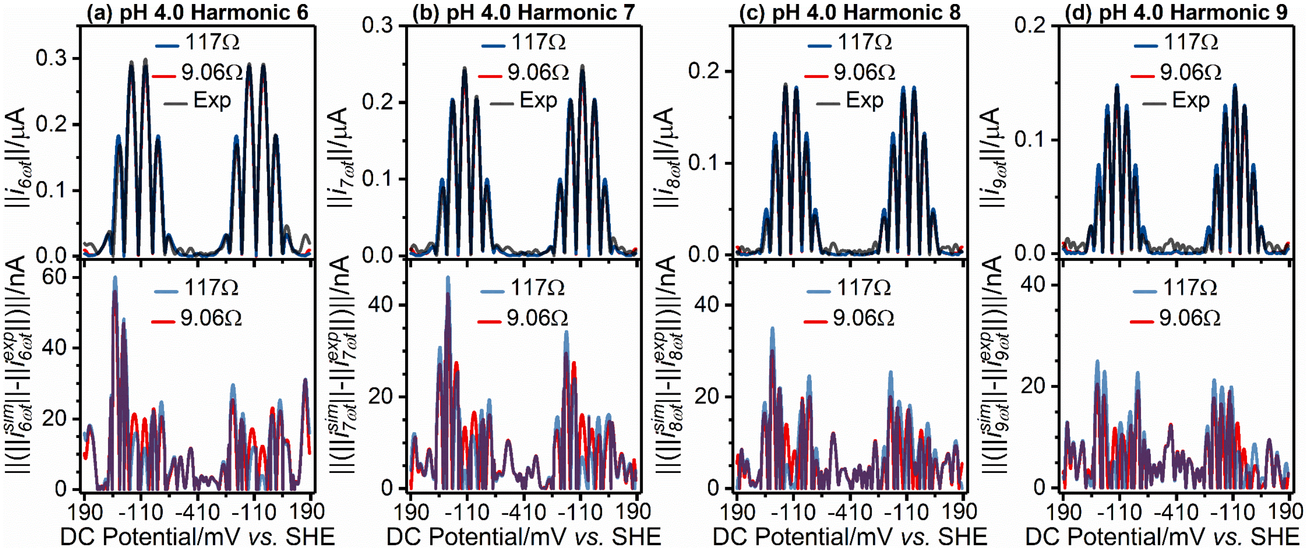

Figure S16 (a-d, top panels) Current-potential plots of harmonics 6 to 9 of (grey line) pH 4.0 experiment 3 ("Exp") data and best fit simulated data using (red line) a concerted two-electron (n_3 = 2) electron transfer model and (blue line) a concerted two-electron using best fit parameters but with an uncompensated resistance of 117**Ω**. (Bottom panels) Residual current-potential plots corresponding to the panel above. The parameter values used to generate the simulations are reported in Table 1 and Table S3.

14 References

Adamson, H., Bond, A.M., and Parkin, A. (2017a). Probing biological redox chemistry with large amplitude Fourier transformed ac voltammetry. *Chemical Communications* 53(69), 9519-9533. doi: 10.1039/C7CC03870D.

Adamson, H., Robinson, M., Bond, P.S., Soboh, B., Gillow, K., Simonov, A.N., et al. (2017b). Analysis of HypD Disulfide Redox Chemistry via Optimization of Fourier Transformed ac Voltammetric Data. *Analytical Chemistry* 89(3), 1565-1573. doi: 10.1021/acs.analchem.6b03589.

Andrieu, C., and Thoms, J. (2008). A tutorial on adaptive MCMC. *Statistics and Computing* 18(4), 343-373. doi: 10.1007/s11222-008-9110-y.

Bard, A.J., and Faulkner, L.R. (2000). *Electrochemical Methods: Fundamentals and Applications, 2nd Edition.* John Wiley & Sons, Incorporated.

Bond, A.M. (2002). *Broadening Electrochemical Horizons: Principles and Illustration of Voltammetric and Related Techniques.* Oxford University Press.

Clerx, M., Robinson, M., Lambert, B., Lei, C.L., Ghosh, S., Mirams, G.R., et al. (2019). Probabilistic Inference on Noisy Time Series (PINTS). *Journal of Open Research Software* 7(1), Article 23. doi: http://doi.org/10.5334/jors.252.

Gavaghan, D.J., Cooper, J., Daly, A.C., Gill, C., Gillow, K., Robinson, M., et al. (2018). Use of Bayesian Inference for Parameter Recovery in DC and AC Voltammetry. *ChemElectroChem* 5(6), 917-935. doi: https://doi.org/10.1002/celc.201700678.

Gundry, L., Guo, S.-X., Kennedy, G., Keith, J., Robinson, M., Gavaghan, D., et al. (2021). Recent advances and future perspectives for automated parameterisation, Bayesian inference and machine learning in voltammetry. *Chemical Communications*. doi: 10.1039/D0CC07549C.

Haario, H., Saksman, E., and Tamminen, J. (2001). An adaptive Metropolis algorithm. *Bernoulli* 7(2), 223-242, 220.

Harris, C.R., Millman, K.J., van der Walt, S.J., Gommers, R., Virtanen, P., Cournapeau, D., et al. (2020). Array programming with NumPy. *Nature* 585(7825), 357-362. doi: 10.1038/s41586-020-2649-2.

Hirst, J. (2006). Elucidating the mechanisms of coupled electron transfer and catalytic reactions by protein film voltammetry. *Biochimica et Biophysica Acta (BBA) - Bioenergetics* 1757(4), 225-239. doi: https://doi.org/10.1016/j.bbabio.2006.04.002.

Lambert, B. (2018). *A Student's Guide to Bayesian Statistics.* SAGE Publications.

Lee, C.-Y., Stevenson, G.P., Parkin, A., Roessler, M.M., Baker, R.E., Gillow, K., et al. (2011). Theoretical and experimental investigation of surface-confined two-center metalloproteins by large-amplitude Fourier transformed ac voltammetry. *Journal of Electroanalytical Chemistry* 656(1), 293-303. doi: https://doi.org/10.1016/j.jelechem.2010.08.012.

Stevenson, G.P., Lee, C.-Y., Kennedy, G.F., Parkin, A., Baker, R.E., Gillow, K., et al. (2012). Theoretical Analysis of the Two-Electron Transfer Reaction and Experimental Studies with Surface-Confined Cytochrome c Peroxidase Using Large-Amplitude Fourier Transformed AC Voltammetry. *Langmuir* 28(25), 9864-9877. doi: 10.1021/la205037e.

Virtanen, P., Gommers, R., Oliphant, T.E., Haberland, M., Reddy, T., Cournapeau, D., et al. (2020). SciPy 1.0: fundamental algorithms for scientific computing in Python. *Nature Methods* 17(3), 261-272. doi: 10.1038/s41592-019-0686-2.
